# Kinesin‐Induced Buckling Reveals the Limits of Microtubule Self‐Repair

**DOI:** 10.1002/advs.202521721

**Published:** 2026-03-12

**Authors:** Shweta Nandakumar, Jonas Bosche, Mirko Wieczorek, Constantin Matteo Albrecht, Belinda König, Mona Grünewald, Ludger Santen, Stefan Diez, Reza Shaebani, Laura Schaedel

**Affiliations:** ^1^ Experimental Physics and Center for Biophysics Saarland University Saarbrücken Germany; ^2^ Theoretical Physics and Center for Biophysics Saarland University Saarbrücken Germany; ^3^ B CUBE ‐ Center for Molecular Bioengineering TUD Dresden University of Technology Dresden Germany; ^4^ Max Planck Institute of Molecular Cell Biology and Genetics Dresden Germany; ^5^ Cluster of Excellence Physics of Life TUD Dresden University of Technology Dresden Germany; ^6^ PharmaScienceHub (PSH) Saarbrücken Germany

**Keywords:** cytoskeleton, kinesin, microtubules, molecular motors

## Abstract

Microtubules are stiff cytoskeletal polymers whose ability to rapidly switch between growth and disassembly relies on a metastable lattice. This metastability is also reflected in their sensitivity to environmental conditions and in intrinsic lattice dynamics, where spontaneous tubulin loss is balanced by tubulin incorporation from solution—a process that also enables microtubules to self‐repair when damaged. Whether such intrinsic self‐repair is sufficient to preserve microtubule integrity during dynamic molecular motor‐induced buckling, which frequently occurs in cells, remains unclear. Here, we show that kinesin‐driven microtubule buckling in vitro induces severe lattice damage, leading to extensive tubulin incorporation. In many cases, however, the damage exceeds the microtubules’ capacity for self‐repair, resulting in breakage. In contrast, microtubules survive continuous buckling substantially longer in the presence of intracellular factors. Our results identify the limits of intrinsic microtubule self‐repair and demonstrate that additional cellular mechanisms are essential to maintain microtubule integrity under sustained mechanical load.

## Graphical abstract

This study shows that kinesin‐driven buckling induces extensive microtubule lattice damage that often exceeds intrinsic self‐repair and leads to filament failure. While curvature, motor motility, and force individually cause limited damage, their combination overwhelms repair. Intracellular factors enhance microtubule resilience under sustained load, suggesting that cells actively tune microtubule mechanical responses.

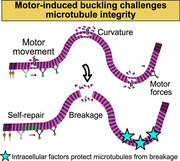

## 1 Introduction

Microtubules are highly dynamic cytoskeletal filaments that support intracellular organization and adapt rapidly to physiological cues [1]. Microtubule tip dynamics—alternating between growth and disassembly—is driven by GTP hydrolysis [1, 2], which renders the microtubule lattice inherently metastable and particularly sensitive to environmental conditions such as temperature [3, 4]. This inherent fragility not only enables microtubules to remain responsive to signals, but also makes them vulnerable to destabilization.

A manifestation of this metastability is intrinsic lattice dynamics, wherein tubulin subunits dissociate from the lattice, particularly at structural defects, and are replaced by free tubulin from solution [5]. This process occurs slowly but can be accelerated by repeated bending via orthogonal fluid flow [6], the activity of severing enzymes and the non‐enzymatic microtubule‐associated protein (MAP) tau [7, 8], and even the translocation of unloaded motor proteins [9, 10, 11]. Although other MAPs such as CLASP [12] and CLIP‐170 [13] may support repair or stabilization, it is generally assumed that microtubules have sufficient intrinsic self‐repair capacity to withstand damage [6, 14, 15]. However, in cells, microtubules are frequently exposed to strong and dynamic deformations [16, 17], including pronounced buckling caused by opposing motor forces and anchorage points within the cytoplasm [18, 19, 20, 21, 22, 23, 24]. Whether intrinsic self‐repair is sufficient to maintain microtubule integrity under such sustained mechanical stress is unclear.

Here, we use a combination of in vitro reconstitution, a stochastic computational model, and supporting cellular data to investigate microtubule behavior under motor‐induced buckling. We find that buckling leads to accelerated lattice damage and extensive tubulin incorporation, and that damage frequently exceeds the capacity of self‐repair, resulting in microtubule breakage. However, in the presence of intracellular factors, microtubules are significantly more resilient. These findings reveal the limits of intrinsic microtubule self‐repair and highlight the essential contribution of cellular mechanisms in preserving microtubule integrity under persistent mechanical load.

## 2 Results

### 2.1 Static Curvature Induces Microtubule Self‐Repair

**FIGURE 1:**
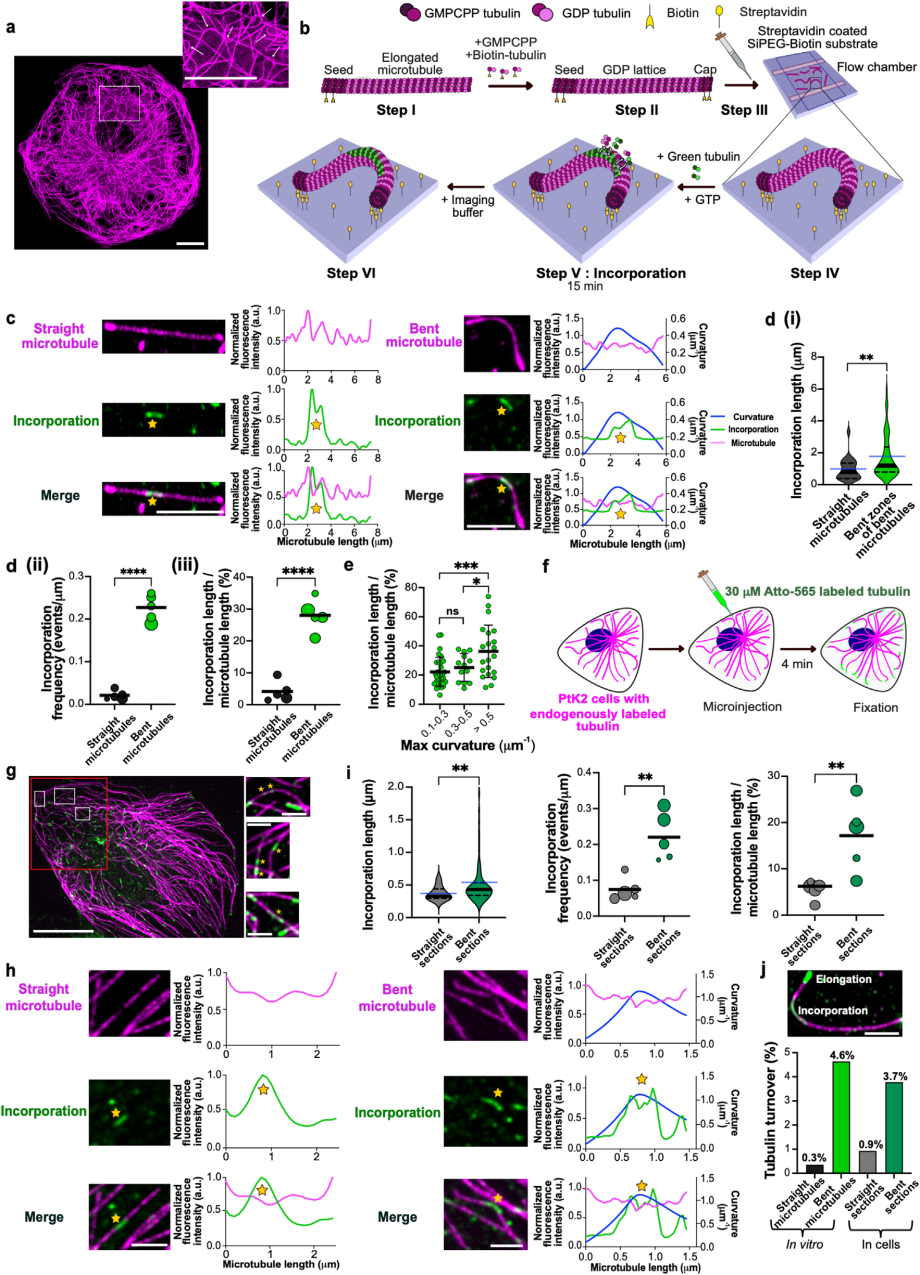
Static curvature triggers microtubule damage and consequent self‐repair. (a) Microtubules adopt bent and buckled conformations in cells: Image of bent microtubules in a PtK2 cell (tubulin‐eGFP, represented in magenta). Inset shows a zoomed‐in image with white arrows indicating bent microtubules. Scale bars: 10 µm. (b) Schematic of the in vitro experimental setup used to assess self‐repair in static bent microtubules. Capped GDP microtubules (shown in magenta) with biotinylated GMPCPP seeds and caps (Step I and II) were flushed onto a streptavidin coated flow chamber made from SiPEG‐biotin passivated cover‐glasses (Step III). Microtubules of various curvatures were obtained by alternating the flow direction (Step IV). Microtubules were then incubated with 5 µM green labeled tubulin for 15 min (Step V) followed by washing with imaging buffer (Step VI). (c) Example images showing incorporation of green labeled tubulin (marked with a yellow star) in a straight and in a bent microtubule in vitro. Scale bar: 5 µm. Graphs represent line scans of the microtubule (magenta), the incorporation channel (green) and curvature (blue). Profiles have been normalized to 1 for the maximum value of the intensity (a.u.) for the microtubule and incorporation channel, respectively (d,i): Violin plot showing longer incorporation stretches in zones of high local curvature in bent microtubules in vitro. Total length of microtubules analyzed: 1104 µm from three independent experiments (*p* = 0.0069 using Mann‐Whitney test) (n = 23 incorporations for straight microtubules, n = 83 incorporations for bent microtubules). Black solid line represents the median and dotted lines represent the interquartile range. Blue line represents the mean. (ii): Bubble plot showing higher frequency of incorporations in bent microtubules when compared to straight microtubules in vitro. Bubble sizes scale with the total microtubule length analyzed. Each circle represents an independent dataset (comprising of 291, 295, 350, 165, and 65 µm of total microtubule length analyzed for bent microtubules and 311, 347, 340, 230, and 366 µm of total microtubule length analyzed for straight microtubules). The black line represents the mean. *p* < 0.0001 using unpaired *t*‐test. (iii): Bubble plot showing higher amount of lattice turnover, estimated as incorporation length/ microtubule length, in bent microtubules when compared to straight microtubules in vitro. Bubble sizes scale with the total microtubule length analyzed. Each circle represents an independent dataset (comprising 291, 295, 350, 165, and 65 µm of the total microtubule length analyzed for bent microtubules and 311, 347, 340, 230, and 366 µm of the total microtubule length analyzed for straight microtubules). The black line represents the mean. *p* < 0.0001 using unpaired *t*‐test. (e) Scatter dot plot comparing the lattice length with incorporation in bent microtubules across different curvature ranges in vitro. Total length of microtubules analyzed: 1104 µm from three independent experiments. Black lines represent the mean and S.D. *p* = 0.3597 (not significant; for curvatures 0.1–0.3 and 0.3–0.5 µm^−1^), *p* = 0.0006 (for curvatures > 0.5 and 0.1–0.3 µm^−1^) and *p* = 0.0493 (for curvatures > 0.5 µm^−1^ and 0.3–0.5 µm^−1^) using unpaired *t*‐test. (f) Schematic of experimental setup used by Gazzola et al., 2023 to assess microtubule self‐repair in PtK2 cells. 30 µM of ATTO‐565 labeled tubulin (represented here in green) was microinjected into PtK2 cells expressing endogenous tubulin‐eGFP (represented here in magenta) and the cells were fixed after 4 min and imaged. (g) Example images showing microtubule self‐repair in a PtK2 cell, scale bar: 20 µm. Box with red outline indicates a selected region of interest. Insets show zoomed‐in images of microtubule sections with incorporations marked with a yellow star. Scale bars of inset images: 2 µm. (h) Self‐repair (marked with a yellow star) in both straight and bent microtubule sections in cells. Scale bar: 2 µm. Graphs represent line scans of the microtubule (magenta), curvature (blue) and the incorporation channel (green). Profiles have been normalized to 1 for the maximum value of the intensity (a.u.) for the microtubule and incorporation channel. (i) Left: Violin plot showing longer incorporation stretches in bent microtubule sections in cells. Total length of microtubules analyzed: 401.96 µm from five cells (*p* = 0.0025 using Mann‐Whitney test.) (n = 37 incorporations for straight sections and n = 84 incorporations for bent sections). Black line represents the median and dotted lines represent the interquartile range. Blue line represents the mean. Center: Bubble plot showing higher frequency of incorporations in bent microtubule sections when compared to straight microtubule sections in cells. Bubble sizes scale with the total microtubule length analyzed. Each circle represents an independent dataset from one cell (comprising of 130, 98, 57, 44, and 72 µm of total length analyzed for bent sections and 22, 56, 74, 36, and 153 µm of total microtubule length analyzed for straight sections. Black line represents the mean. *p* = 0.0022 using unpaired *t*‐test. Right: Bubble plot showing higher amount of lattice length with incorporation estimated as incorporation length/microtubule length, in bent microtubule sections when compared to straight microtubule sections in cells. Bubble sizes scale with the total microtubule length analyzed. Each circle represents an independent dataset from one cell (comprising of 130, 98, 57, 44, and 72 µm of total length analyzed for bent sections and 22, 56, 74, 36, and 153 µm of total microtubule length analyzed for straight sections). Black lines represent the mean. *p* = 0.0099 using unpaired *t*‐test. (j) Top: Example image showing an incorporation stretch and the elongated tip that was used as a reference to estimate the amount of lateral tubulin incorporation (see Methods). Scale bar: 2 µm. Bottom: Higher % of tubulin turnover in bent microtubules, both in cells and in vitro. Three independent experiments were analyzed for each condition. n = 21 incorporations for straight microtubules (in vitro), n = 51 incorporations for bent microtubules (in vitro), n = 16 incorporations for straight sections (cells) and n = 32 incorporations for bent sections (cells). Refer methods for estimation of % tubulin turnover and Supplementary Figure S1e,f for estimation of amount of lateral tubulin incorporation.

Despite their high flexural rigidity [25], microtubules frequently adopt curved conformations in cells [26, 27, 28], suggesting that they are exposed to considerable intracellular forces. For example, in fixed PtK2 cells with endogenously labeled tubulin, we observed that many microtubules display locally highly curved regions (Figure 1a). This raises the question whether static bending promotes lattice damage and repair. To test this, we reconstituted statically curved microtubules in vitro (Figure 1b, see Methods): First, we polymerized dynamic microtubules from stabilized biotinylated seeds (step I) and stabilized their ends with biotin‐tubulin caps (step II). Importantly, the GDP microtubule lattice between seed and cap was not stabilized. These microtubules were then introduced into streptavidin‐coated flow chambers assembled from passivated cover glasses (step III), where the biotinylated ends attached to the substrate. We alternated the flow direction during chamber loading, frequently resulting in microtubules adopting bent conformations upon immobilization at both ends (step IV). The resulting microtubule curvatures are comparable to those of intracellular microtubules (Supplementary Figure S1d). Next, we exposed these microtubules to soluble fluorescently labeled tubulin for 15 min to allow for incorporation (step V), followed by washout and imaging (step VI). We observed tubulin incorporation both in straight and bent microtubules, where incorporation events appear as localized stretches along the microtubule lattice (Figure 1c, Supplementary Figure S1a). While the length of individual incorporation stretches does not differ between bent and straight microtubules when analyzed along the entire microtubule (Supplementary Figure S1b), focusing specifically on bent zones (see curvature analysis section in the Methods) reveals a modest increase in incorporation length compared to straight microtubules (Figure 1d(i)). In contrast, the spatial frequency of incorporation events is substantially higher in bent microtubules (Figure 1d(ii)), resulting in 28% of lattice lengths showing tubulin incorporation, compared to only 4% in straight microtubules (Figure 1d(iii)). When grouping microtubules by curvature, we found a progressive increase in tubulin incorporation with increasing curvature (Figure 1e). These observations suggest that static bending promotes lattice damage and subsequent self‐repair in vitro.

We then proceeded to assess the relationship between curvature and tubulin incorporation in cells by reanalyzing a previously published dataset [29] of self‐repair in PtK2 cells expressing endogenously GFP‐tagged tubulin (represented here in magenta). These cells were micro‐injected with 30 µM purified, Atto‐565 labeled tubulin (represented here in green), followed by fixation after 4 min of incubation (Figure 1f,g). Since we rarely observed globally straight microtubules in PtK2 cells, we decided to analyze microtubules section‐wise (see Methods). Similar to our observations in vitro, we detected incorporation events along both straight and bent microtubule sections (Figure 1h, Supplementary Figure S1c). Tubulin incorporation stretches are longer (Figure 1i, left) and more frequent (Figure 1i, center) in bent microtubule sections compared to straight microtubule sections, overall leading to a larger proportion of lattice lengths with incorporations (Figure 1i, right), reminiscent of our in vitro results.

Since the fluorescence signal of incorporation stretches in intracellular microtubules appears more intense compared to in vitro microtubules (suggesting a higher fraction of the lattice has been replaced at these sites), we then estimated the local amount of incorporated tubulin across protofilaments in vitro and in cells (see Methods). For this, we normalized the intensities of the incorporation stretches to the intensities of microtubule tips grown with tubulin of the same color, which we presume to consist of 13 protofilaments (Figure 1j, top). This quantification serves as a direct readout of the lateral extent of tubulin incorporation, revealing that incorporation typically occurs over 1–3 protofilaments, occasionally reaching up to 9 protofilaments in cells (See Methods, Supplementary Figure S1e,f). Thus, in most cases, only a small portion of the lattice is exchanged. Together with the mean length of lattice showing incorporation (Figure 1d(iii) and Figure 1i, right), this analysis allowed us to estimate the total amount of tubulin turnover in each condition by accounting for both the longitudinal extent of incorporation along the microtubule and its lateral spread across protofilaments. Figure 1j (bottom) shows that tubulin turnover is more pronounced in bent as opposed to straight microtubules, both in vitro and in cells, yet remains below 5%. Together, these data show that microtubule curvature leads to increased tubulin incorporation both in vitro and in cells. While previous work has shown that repeated bending cycles by orthogonal fluid flow can induce microtubule damage [6], our findings demonstrate that sustained static curvature is sufficient to enhance tubulin incorporation, indicating a direct mechanical contribution to lattice damage and repair.

### 2.2 In Vitro Reconstitution of Cell‐Like Microtubule Buckling

**FIGURE 2:**
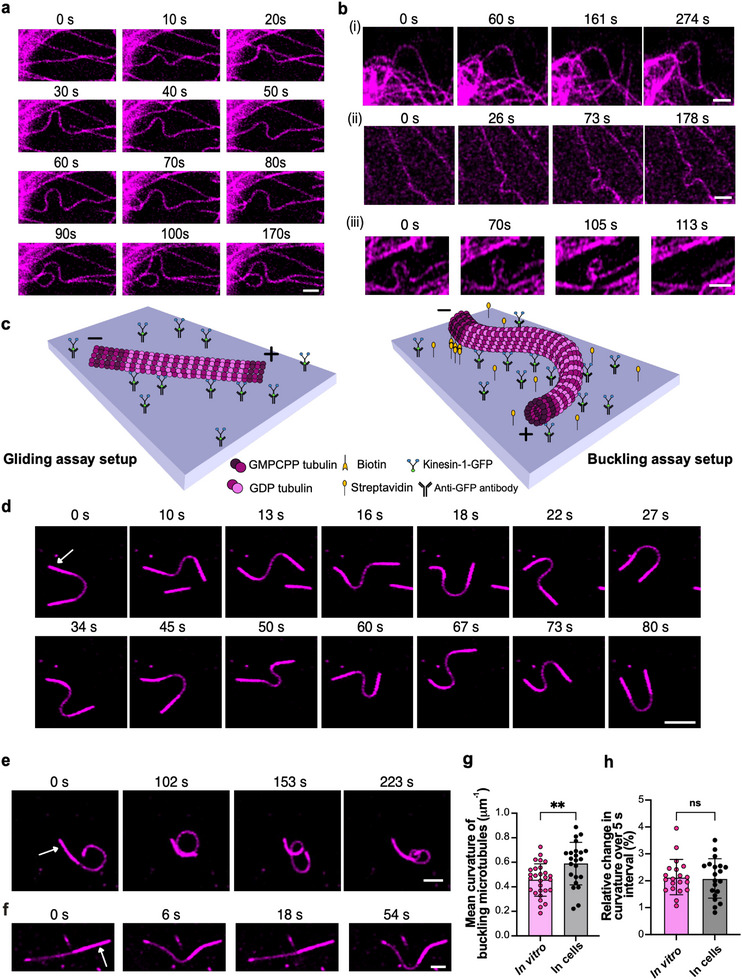
In vitro reconstitution of cell‐like microtubule buckling. (a) Time‐lapse sequence showing change in local microtubule curvature over time in endogenously labeled PtK2 cells (tubulin‐eGFP, represented in magenta). Scale bar: 2 µm. (b) Time‐lapse sequence of microtubule bending and buckling events in PtK2 cells: (i) curved microtubule persisting in a bent form; (ii) dynamically buckling microtubule; (iii) breakage of a looping microtubule. Scale bars: 2 µm. (c) Schematic of the experimental setup used to reconstitute buckling in vitro. In typical gliding assays (left), kinesin‐1‐GFP motor proteins are immobilized onto an anti‐GFP antibody coated glass surface and GDP microtubules glide over the layer of motors in the presence of ATP. In the buckling assay setup (right), the surface is coated with equal amounts of streptavidin and anti‐GFP antibody. Using capped microtubules with seeds containing biotin, we immobilized one end (minus end) to the surface. When the mix with ATP is added, microtubules buckle dynamically. (d) Time‐lapse sequence of a microtubule displaying regular flagella‐like oscillations. White arrow in the first frame indicates the position of the microtubule seed (minus end). Scale bar: 5 µm. (e) Time‐lapse sequence of a looping microtubule. White arrow in the first frame indicates the position of the microtubule seed (minus end). Scale bar: 2 µm. (f) Microtubule showing a regular beating pattern. White arrow in the first frame indicates the position of the microtubule seed (minus end). Scale bar: 2 µm. (g) Comparison of mean curvature of buckling microtubules in cells and in vitro (n = 28 timepoints, in vitro from 4 buckling microtubules from three independent experiments and n = 23 timepoints from 4 cells analyzed from 2 independent experiments). *p* = 0.0032 using unpaired *t*‐test. Error bars represent the S.D. (h) Comparison of rate of change in curvature in buckling microtubules in 5 s in cells and in vitro (n = 20 from three independent experiments for in vitro and from 5 cells analyzed from 2 independent experiments). *p* = 0.8125 (not significant; ns), using unpaired *t*‐test. Error bars represent the S.D.

While the striking similarity between tubulin incorporation in vitro and in PtK2 cells highlights the role of microtubule curvature, our in vitro observations were made after an incubation time three times longer than that used in PtK2 cells (15 min vs. 4 min). This discrepancy suggests that additional factors contribute to microtubule damage and repair in PtK2 cells. When examining dynamic microtubules in live cells, it becomes apparent that local microtubule curvature often changes over time (Figure 2a; Supplementary Movie S1), as has been described earlier [18]. While some microtubules maintain a curved conformation with little change over extended periods (Figure 2b(i)), many exhibit dynamic buckling on short timescales (Figure 2b(ii)). In rare instances, we observed microtubule breakage, typically occurring in regions of high curvature (Figure 2b(iii), Supplementary Figure S3b; see Supplementary Figure S3a for quantification of different microtubule bending events in cells). While microtubule phenotype and the proportion of bending and buckling events may differ across cell types and stages of the cell cycle, existing literature suggests that this is a common feature among cell types. Our observations (Figure 2b) and quantifications of microtubule deformation events (Supplementary Figure S3a) and microtubule curvature in PtK2 cells (Figure 2g) are consistent with observations reported in fibroblasts [27], LLC‐PK1 epithelial cells [17], contracting cardiomyocytes [30], neuronal NG105‐18 cells [31] and *Xenopus* melanophores [19]. These cell‐based observations prompted us to investigate how dynamic microtubule buckling affects microtubule integrity.

Since the origin and magnitude of intracellular forces are difficult to determine, we sought to reconstitute microtubule buckling in vitro. Given that the dynamic, short‐wavelength microtubule buckling in cells has been mainly attributed to molecular motor activity [17, 23, 26], we adapted a motor‐based microtubule gliding assay setup to mimic this behavior (Figure 2c) [32, 33, 34]. We first grew dynamic microtubules from biotinylated seeds and stabilized their ends with biotin‐free GMPCPP‐tubulin (see Methods). We then introduced these microtubules into flow chambers coated with streptavidin and GFP‐labeled kinesin‐1 immobilized via anti‐GFP antibodies. All assays used purified kinesin‐1 (heavy chain truncated to 560 aa, see Methods). Since the biotinylated seeds typically elongate at their plus ends, microtubules are effectively anchored at their minus ends to the streptavidin coated surface. When ATP is added, motor‐driven translocation of the microtubule shaft leads to a build‐up of axial compressive forces, thereby inducing microtubule buckling.

In our in vitro buckling assay, microtubules exhibit a range of dynamic deformation modes (Figure 2d–f; Supplementary Movie S2). To describe this diversity more systematically, we distinguish three recurring deformation patterns: some microtubules show regular, flagella‐like oscillations (Figure 2d), characterized by a repeating motion pattern with regions of high curvature that originate near the anchor point and travel toward the free end (see Supplementary Movie S3). Others form chaotic loops without any apparent repeating pattern (Figure 2e). Some also display a beating‐like motion (Figure 2f), which also repeats at regular intervals but lacks traveling regions of high curvature, and intermittently returns to relatively straight conformations.

To compare these deformation dynamics to those observed in PtK2 cells, we measured microtubule curvatures and rates of curvature change in both systems. The distributions are similar, with slightly higher curvatures in cells, indicating that our assay reproduces the dynamic shape fluctuations of buckling microtubules seen in cells (Figure 2g,h), and reaches curvatures comparable to those of the bent zones in static bent microtubules (Supplementary Figure S3c). While the precise intracellular force patterns remain elusive, these observations suggest that key aspects of motor‐induced buckling can be reconstituted using a simplified in vitro system.

### 2.3 Kinesin‐Driven Buckling Causes Extensive Damage and Self‐Repair

**FIGURE 3:**
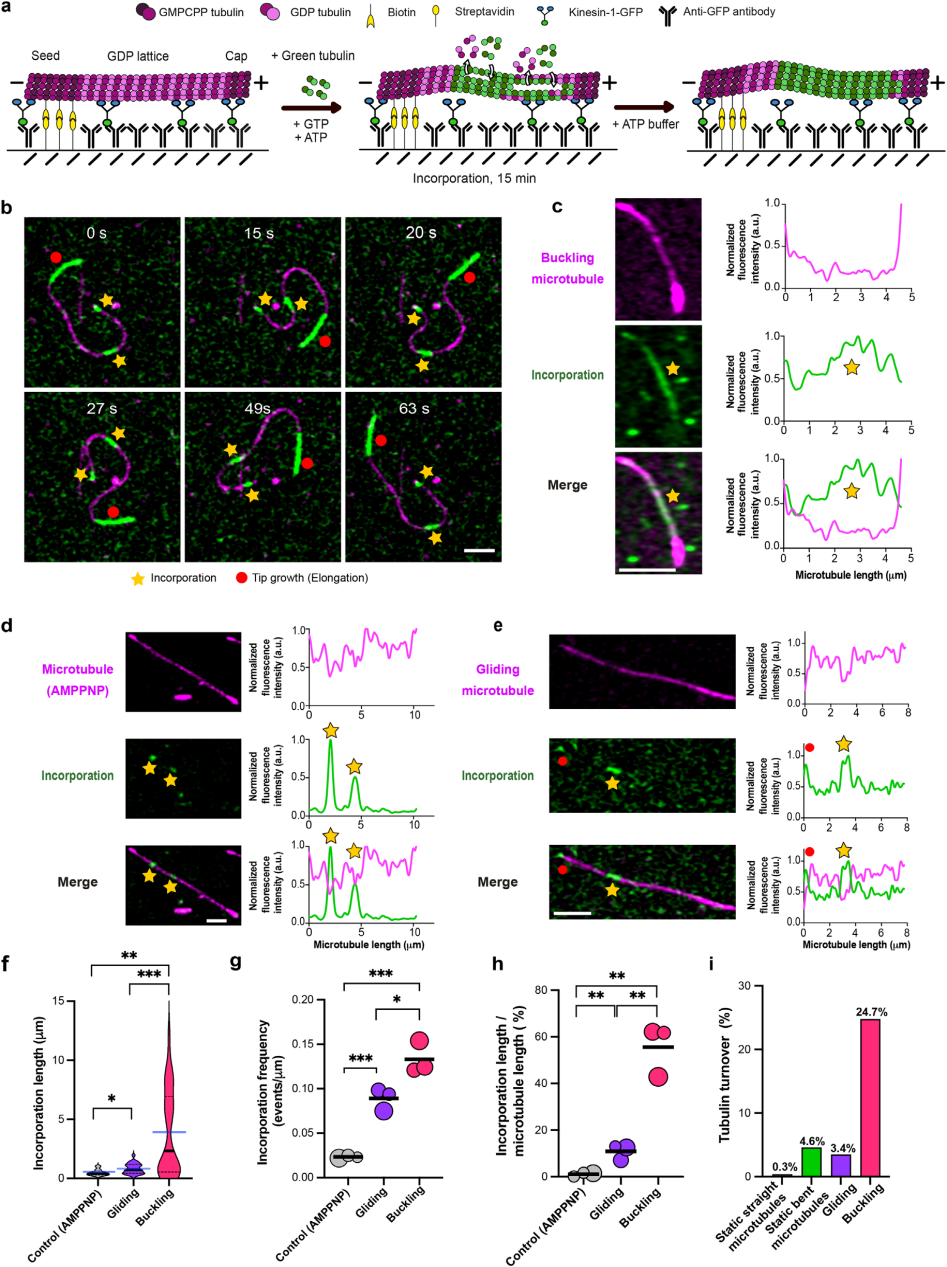
Extensive damage and consequent self‐repair in buckling microtubules. (a) Schematic of the experimental setup used to assess self‐repair in buckling microtubules in vitro. (b) Time‐lapse sequence of a buckling microtubule showing incorporations of green labeled tubulin (marked with a yellow star) and the elongated tip (highlighted with a red circle). Scale bar: 2 µm. (c) Example image showing incorporation of green labeled tubulin (marked with a yellow star) in a buckling microtubule. Scale bar: 2 µm. Graphs represent line scans of the microtubule (magenta) and the incorporation channel (green). Profiles have been normalized to 1 with the maximum value of the intensity (a.u.) of the microtubule and incorporation channel, respectively. Scale bar: 2 µm. (d) Example image showing incorporation of green labeled tubulin (marked with a yellow star) in the buckling setup with static motors using AMPPNP. Graphs represent line scans of the microtubule (magenta) as well as the incorporation channel (green). Profiles have been normalized to 1 with the maximum value of the intensity (a.u.) of the microtubule and incorporation channel, respectively. Scale bar: 2 µm. (e) Example image showing incorporation of green labeled tubulin (marked with a yellow star) in a gliding microtubule. Elongation of the microtubule tip is highlighted with a red circle. Graphs represent line scans of the microtubule (magenta) as well as the incorporation channel (green). Profiles have been normalized to 1 with the maximum value of the intensity (a.u.) of the microtubule and incorporation channel, respectively. Scale bar: 2 µm. (f) Violin plots showing extensive incorporations in buckling microtubules. Total length of microtubules analyzed: 567 µm for gliding, 474 µm for buckling and 586 µm for control‐AMPPNP from three independent experiments per condition. Black line represents the median and dotted lines represent the interquartile range. Blue lines represent the mean for each condition. *p =* 0.0018 (buckling‐control), *p =* 0.0172 (gliding‐control), and *p =* 0.0005 (gliding‐buckling) using Mann‐Whitney test (n = 9 incorporations for control‐AMPPNP, n = 52 incorporations for gliding and n = 36 incorporations for buckling). (g) Bubble plot showing a higher frequency of incorporations in buckling microtubules. Bubble sizes scale with the total microtubule length analyzed. Each circle represents an independent experiment (comprising of 226, 158, and 183 µm of total microtubule length analyzed for gliding; 142, 171, and 161 µm of total microtubule length analyzed for buckling and 218, 173, and 195 µm of total microtubule length analyzed for control‐AMPPNP). Black line represents the mean. *p =* 0.0261 (for gliding‐buckling); *p* = 0.0008 (gliding‐control); *p* = 0.0005 (buckling‐control) using unpaired‐*t*‐test. (h) Bubble plot showing a higher amount of lattice length with incorporation, estimated as incorporation length/ microtubule length, in buckling microtubules in vitro. Bubble sizes scale with the total microtubule length analyzed. Each circle represents an independent experiment (comprising of 226, 158 and 183 µm of total microtubule length analyzed for gliding; 142, 171, and 161 µm of total microtubule length analyzed for buckling and 218, 173, and 195 µm of total microtubule length analyzed for control‐AMPPNP). Black lines represent the mean. *p* = 0.0076 (gliding‐control); *p* = 0.0010 (buckling‐control) and *p* = 0.0026 (gliding‐buckling) using unpaired *t*‐test. (i) Higher % of tubulin turnover in buckling microtubules. Total length of microtubules analyzed: 567 µm for gliding, 474 µm for buckling and 586 µm for control‐AMPPNP from three independent experiments per condition. Refer methods for estimation of % tubulin turnover and Supplementary Figure S7e for estimation of amount of lateral tubulin incorporation.

We then used our in vitro assay to study microtubule damage and self‐repair in buckling microtubules (Figure 3a). For this, we let capped GDP microtubules (magenta) buckle dynamically in the presence of free tubulin (green) for 15 min before washing out the labeled free tubulin and imaging. Figure 3b shows an example image sequence of a buckling microtubule after washout. Buckling microtubules exhibit up to 12 µm long tubulin incorporation stretches that frequently extend along a large part of the microtubule lattice (Figure 3c,f; Supplementary Movies S5 and S6). In contrast, when keeping microtubules statically attached to motors using the non‐hydrolysable ATP‐analogue AMPPNP, we only observed tubulin incorporation stretches of less than 2 µm length (Figure 3d,f). In gliding microtubules grown from biotin‐free seeds (see Methods), incorporation stretches also appear much shorter than in buckling microtubules (Supplementary Movie S4), although they are longer than in static microtubules (Figure 3e,f), consistent with previous reports [9, 10, 11]. The spatial incorporation frequency also increases from AMPPNP to gliding and buckling microtubules, though the difference in incorporation frequency between buckling microtubules and the other two cases is less pronounced (Figure 3g). This may be due to overlapping incorporations that cannot be distinguished in our analysis, since buckling microtubules exhibit very long incorporation stretches. Overall, we observed tubulin incorporation along 60% of the microtubule lattice length in buckling microtubules (Figure 3h), five times as much as in gliding microtubules and 50 times as much as in static microtubules. Quantification of the total amount of tubulin turnover by accounting for both the longitudinal extent and the lateral spread of incorporation (Supplementary Figure S7e) reveals that buckling microtubules show, by far, the highest turnover (Figure 3i). While even unloaded motors were previously shown to induce microtubule damage and subsequent self‐repair [9, 11, 35, 36], our observations reveal that kinesin‐mediated buckling massively increases this effect.

### 2.4 Limits of Microtubule Self‐Repair Under Kinesin‐Induced Buckling

**FIGURE 4:**
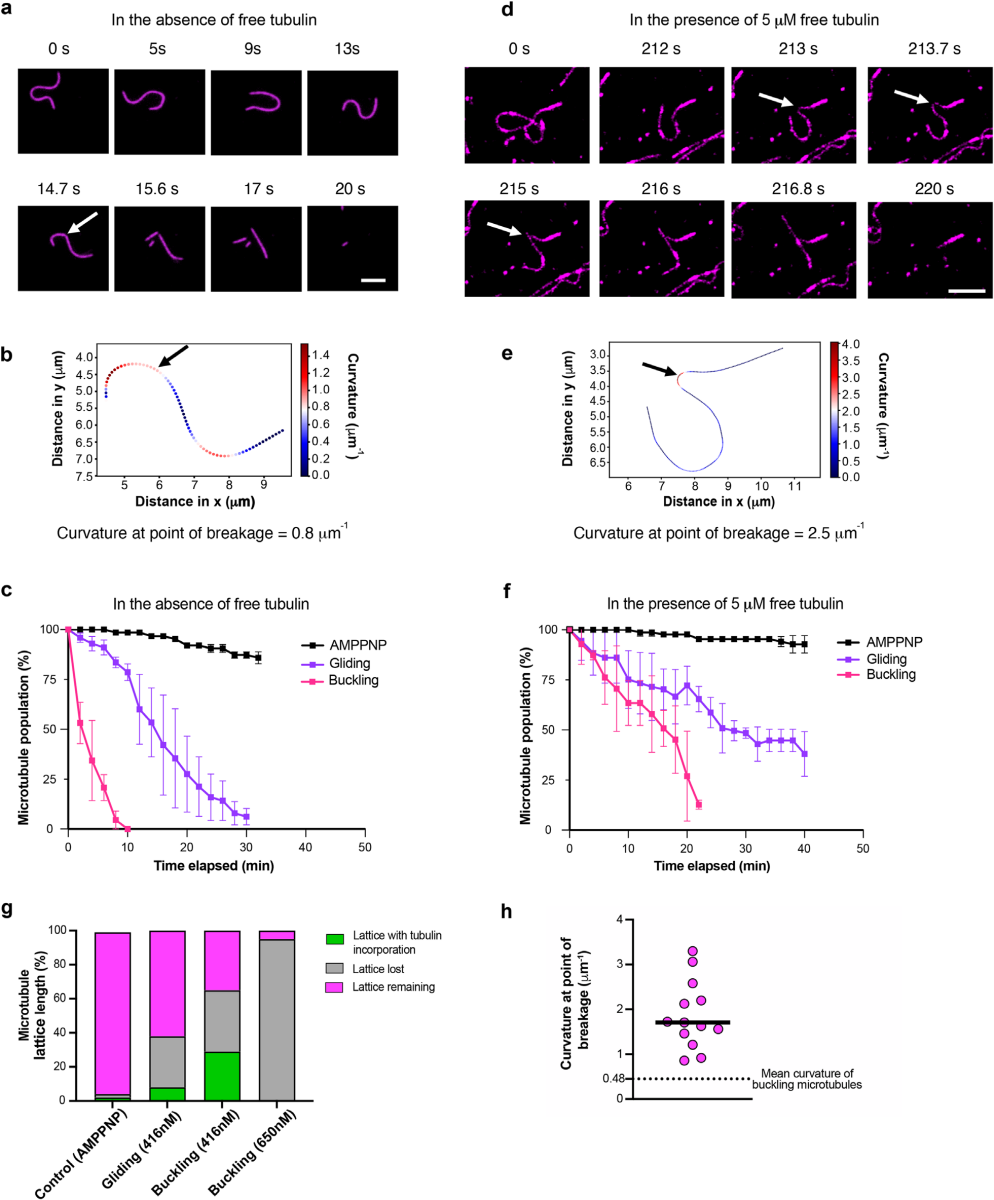
Motor‐induced buckling damages microtubules beyond the limit of self‐repair. (a) Time‐lapse sequence of a buckling microtubule breaking in the absence of free tubulin. White arrow in the frame at 14.7s indicate the point of breakage. Scale bar: 5 µm. (b) Plot showing distribution of local curvature along the microtubule in (a) in the frame (at 14.7s) prior to microtubule breakage. Black arrow indicates point of breakage. (c) Comparison of % microtubule population remaining in control (AMPPNP), gliding and buckling microtubules over time, in the absence of free tubulin. The symbols indicate mean ± S.D. Total length of microtubules analyzed: 567 µm for gliding, 474 µm for buckling and 586 µm for control‐AMPPNP (no: of microtubules analyzed > 100) from two independent experiments per condition. (d) Time‐lapse sequence of a buckling microtubule breaking in the presence of 5 µM free tubulin. Point of breakage indicated by a white arrow. Scale bar: 5 µm. (e) Plot showing distribution of local curvature along the trace of the microtubule in (d) in the frame (at 213.7s) prior to microtubule breakage. Black arrow indicates the point of breakage. (f) Comparison of % microtubule population remaining in control (AMPPNP), gliding and buckling assays over time in the presence of 5 µM free tubulin. The symbols indicate mean ± S.D. Total length of microtubules analyzed: 567 µm for gliding, 474 µm for buckling and 586 µm for control‐AMPPNP (no: of microtubules analyzed >100) from two independent experiments per condition. (g) Bar graph showing the relative proportion of microtubule lattice length lost, repaired (refer to Figure 3g,h) and remaining (refer (f)) at the end of 15 min, in the case of assays with AMPPNP, gliding microtubules (416 nM kinesin), buckling microtubules (416 nM kinesin), and buckling microtubules (650 nM kinesin). No: of microtubules analyzed > 100 from three independent experiments per condition. (h) Curvature of buckling microtubules at point of breakage (n = 13 microtubules from eight independent experiments). Black line represents the median (1.71 µm^−1^). Black dotted line represents the average curvature of buckling microtubules (0.48 µm^−1^; Refer to Figure 2g).

The pronounced tubulin incorporation observed in buckling microtubules suggests that these microtubules experience substantial lattice damage. This leads to the question whether microtubule self‐repair is sufficient to counteract the damage sustained under such dynamic buckling. Since tubulin incorporation is the net outcome of damage and repair, we first assessed microtubule stability in the absence of free tubulin, where microtubules are known to spontaneously disassemble due to gradual tubulin loss even in the absence of external forces [8, 37]. This condition serves as a reference point, removing the possibility of self‐repair and allowing us to directly assess how strongly mechanical stress accelerates microtubule disassembly. We found that buckling microtubules frequently break and disassemble (Figure 4a). Notably, we observed that breakage sites typically coincide with regions of high local curvature (Figure 4b; Supplementary Movie S7), similar to what is seen in cells (Supplementary Figure S3b and in Odde et al., 1999 [27]). After just 4 min, 50% of the buckling microtubules disassemble, and after 10 min, all buckling microtubules disappear (Figure 4c). By contrast, gliding microtubules persist longer, as reported in Triclin et al., 2021 [9]: 50% remain intact after around 14 min, and within 30 min, all gliding microtubules had disassembled. When kinesin motors are rendered static using AMPPNP, microtubule survival is markedly enhanced, with 85% of microtubules remaining intact even after 30 min.

We next asked whether self‐repair via tubulin incorporation could compensate for the damage observed under dynamic buckling (Figure 4d–f). In the presence of free tubulin, gliding microtubules show markedly improved survival compared to the no‐tubulin condition and statically‐bound microtubules experience close to no loss (Figure 4f), consistent with previous reports [9, 11]. In contrast, buckling microtubules still disassemble rapidly, despite the availability of free tubulin (Supplementary Movie S8), typically after breakage in highly curved regions (Figure 4d,e,h; Supplementary Movie S7). After just over 20 min, all buckling microtubules break and disassemble (Figure 4f). Our observations suggest that breakage is more frequent and occurs on shorter timescales than in studies with taxol‐stabilized microtubules [38, 39, 40], which, by design, were performed in the absence of free tubulin and did not account for self‐repair.

To better understand the balance between damage and self‐repair, we compared tubulin incorporation and microtubule survival after 15 min, in the presence of free tubulin across conditions (Figure 4g). Microtubules with static motors (AMPPNP condition) show almost no detectable tubulin incorporation and remain nearly fully intact, indicating minimal lattice damage. In gliding microtubules, 30% of the total initial microtubule length is lost due to breakage, and incorporation stretches appear along 8% of the initial lattice length. Buckling microtubules, driven by the same motor density used for gliding (416 nM), show a greater loss of lattice length (49%) and a much higher fraction of lattice length with incorporations (29%). Strikingly, upon further increasing motor density (650 nM), microtubule lattice damage becomes catastrophic: 95% of the initial length is lost, and no visible tubulin incorporation remains. This indicates that the rate of damage has surpassed the capacity for self‐repair. Interestingly, the median value of curvature at the point of breakage in buckling microtubules is 1.7 µm^−1^ (Figure 4h), closely matching the value of 1.5 µm^−1^ reported by Odde et al., 1999 for occasional microtubule breakage in fibroblasts [27].

Although buckling‐induced microtubule breakage occurs predominantly at regions of high microtubule curvature, we did not observe intermediate conformations (i.e., locally strongly bent or kinked regions) consistent with gradual, localized softening prior to breakage. Instead, continual deformation during buckling is associated with sudden microtubule failure (Figure 4a,b; Supplementary Movies S7 and S8), indicating a rapid transition from deformation to breakage. Consistently, quantification of microtubule curvature over time (Supplementary Movie S3) does not reveal a progressive increase in mean curvature, suggesting that any potential softened states, if present, are too short‐lived to be experimentally resolved under our conditions.

Together, these results highlight the limits of microtubule self‐repair: Statically anchored microtubules remain stable, and gliding microtubules sustain moderate damage that is efficiently repaired, in line with previous reports that self‐repair protects microtubules against breakage from motor motility [9]. In contrast, buckling—especially at high motor density—induces severe lattice disruption that exceeds self‐repair capacity. Thus, dynamic buckling represents a mechanical challenge to microtubule integrity that cannot be balanced by intrinsic self‐repair alone. Based on our experiments, breakage typically occurs in regions of high curvature, suggesting curvature as a determinant of lattice disruption. However, it remains unclear whether these curved regions are also subjected to elevated motor forces, since in our assays we cannot resolve local force patterns along the microtubule.

### 2.5 Worm‐Like Chain Model Recapitulates Microtubule Buckling

**FIGURE 5:**
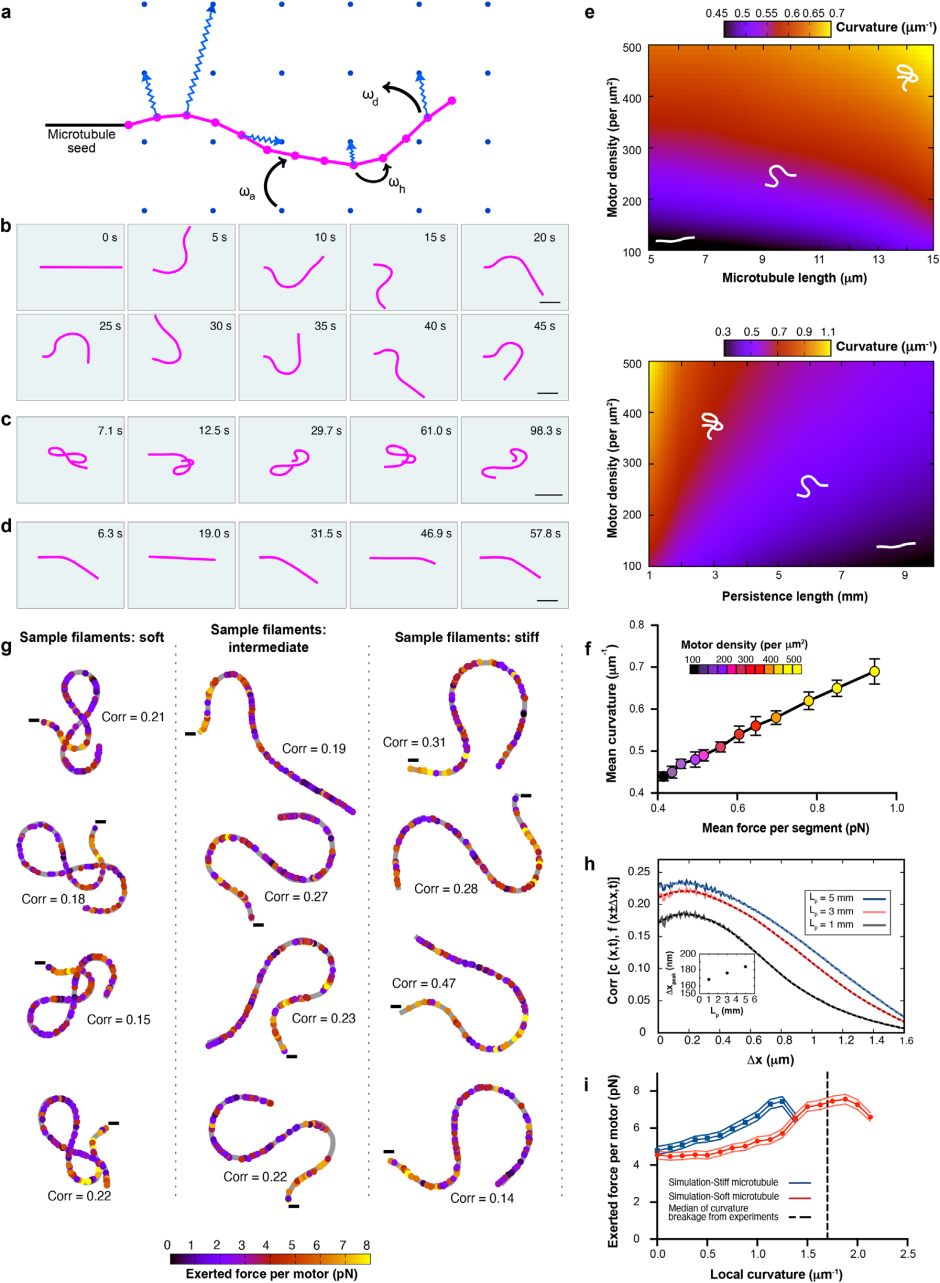
Worm‐like chain model‐based simulations capture microtubule deformation under active forces. (a) Schematic of the simulation setup: motor proteins (represented as blue dots) are uniformly distributed on the substrate and interact with the microtubule by attaching, hopping along it, or detaching with rates ω_a_, ω_h_, and ω_d_, respectively. The minus end of the microtubule (seed, represented in black) is kept fixed. Springs represent pulling forces exerted by attached motors. (b–d) Representative microtubule conformations in different deformation regimes: (b) regular flagella‐like oscillations; (c) loop formation; and (d) regular beating. The motor density is ρ= 248, 494, and 123 µm^−2^, respectively. Scale bars: 2 µm. Other parameters are set to default values mentioned in Supplementary Table S1. (e) Numerical phase diagrams of microtubule deformation under active motor forces. Mean curvature of microtubules as a function of (top) microtubule length and motor density at a fixed persistence length L_p_= 5 mm and (bottom) persistence length and motor density at a fixed microtubule length L= 10 µm. The heat maps display ensemble‐averaged data over 10^5^ filaments per parameter set. The underlying data are discretized into 20 bins along each axis, and a Gaussian smoothing filter was applied to generate the final color map. Schematic white filaments show representative microtubule configurations for corresponding mean curvature values. (f) Mean curvature of the filament versus the mean force exerted on the filament per segment for L = 10 µm, L_p_ = 5 mm, and increasing motor density. The data is ensemble averaged over 10^3^ filaments. (g) Representative microtubule configurations from simulations, color‐coded by the local active force exerted by molecular motors. Each dot represents an attached motor. Shown are examples for three levels of bending rigidity: soft filaments with L_p_ =1 mm (left), intermediate stiffness with L_p_ = 5 mm (middle), and stiff filaments with L_p_ = 10 mm (right). Other parameters: L = 10 µm, ρ = 494 µm^−2^. In each panel, the gray line traces the filament backbone. Colored circles mark the positions of the active motors along the microtubule, with the color indicating the magnitude of the exerted force by each motor. Minus signs denote the fixed ends of the microtubules. 10−15% of the modeled microtubules segments are occupied by motors (refer to Supplementary Figure S6b). Configurations highlight differences in deformation patterns and force localization depending on filament stiffness. For each case, the corresponding curvature‐force correlation is also shown, which is computed at the optimal spatial offset (within the narrow range [0.17, 0.20] µm) and averaged along the filament. (h) Cross‐correlation between the local curvature c(x,t) of a modeled buckling microtubule at position x and time t, and the local force f(x ± ∆x,t) exerted at spatially offset positions at the same time, plotted as a function of spatial offset ∆x (See SI simulation methods for details of Pearson correlation calculation). Results are shown for varying persistence lengths from an ensemble of 10^5^ microtubules. Solid lines represent raw simulation data, while dashed lines indicate smoothed curves obtained using a Savitzky‐Golay filter. Default values for all other parameters are listed in Supplementary Table S1. Cross‐correlation values are averaged over time and along the length of the filament. Inset shows the dependence of the correlation peak position on persistence length (L_p_). (i) Force exerted by each motor versus local curvature, shown for simulated stiff microtubule with L_p_ = 10 mm (dark blue) and soft microtubule with L_p_ = 1 mm (red). Other parameters: L = 5 µm, ρ = 400 µm^−2^. Solid lines represent the mean, and shaded regions indicate standard deviations across the ensemble of 10^5^ microtubules. Vertical dotted line (in black) represents the median curvature of breaking buckling microtubules (1.7 µm^−1^) from Figure 4h.

To better understand the mechanical loads experienced by microtubules in our in vitro buckling assay, we developed a stochastic 2D computational model that captures the key mechanical and dynamic features of our experimental system (Figure 5a; see Supplementary Simulation Methods for a detailed description). We modeled microtubules as worm‐like chains composed of 8‐nm segments—equivalent to the length of a tubulin dimer—the typical step‐size of kinesin [41, 42]. Microtubules are fixed at their minus ends, mimicking the anchored seeds in our experiments. Motors are distributed on a grid at variable densities and modeled as point‐like entities that can bind to the nodes between microtubule segments and step along the microtubule (Figure 5a). Since the motors remain attached to the substrate, their stepping leads to microtubule buckling. The motor parameters—including attachment and detachment rates and force‐dependent stepping kinetics—are based on literature values and our own measurements (see Supplementary Table S1; Supplementary Figure S5a–i; SI Simulation Methods). Unless otherwise indicated, we performed simulations using 10 µm‐long microtubules with 5 mm persistence length (L_p_) and a motor density (ρ) of 400 µm^−2^. Note that the model does not rely on any fit parameters tuned to our experimental system. Rather than aiming for a precise quantitative match, we chose a coarse‐grained model designed to semi‐quantitatively capture essential trends. To verify that the model reproduces our experimental observations both in vitro and in cells, we compared the mean microtubule curvature between simulations, experiments and buckling microtubules in PtK2 cells (Supplementary Figure S6a).

By varying the motor density, we were able to reproduce the distinct dynamic behaviors observed in our experiments: flagella‐like oscillations (Figure 5b, compare to Figure 2d; Supplementary Movie S9), looping (Figure 5c, compare to Figure 2e, Supplementary Movie S10), and beating motions (Figure 5d, compare to Figure 2f; Supplementary Movie S11). We then systematically explored the dependence of microtubule curvature on key parameters by constructing phase diagrams showing the mean microtubule curvature as a function of motor density, microtubule length, and microtubule persistence length (Figure 5e). This reveals intuitive trends: curvature increases with higher motor density and microtubule length and decreases with increasing microtubule stiffness.

To assess how motor‐generated forces influence microtubule shape, we then examined how mean curvature depends on the mean force along the microtubule. Figure 5f shows the mean microtubule curvature as a function of the mean force per microtubule segment at varying motor densities, which effectively increases the number of active motors on the microtubules (Supplementary Figure S6b). Mean curvature scales predictably with the overall applied force.

Next, we aimed to determine which local motor force patterns are responsible for inducing buckling. Figure 5g shows color‐coded force profiles for twelve representative microtubules with different persistence lengths: soft (L_p_ = 1 mm), intermediate (L_p_ = 5 mm) and stiff (L_p_ = 10 mm). Notably, the force distribution is highly heterogeneous and does not consistently coincide with regions of high curvature. When correlating local curvature with the local force per segment, we found only a modest correlation (r ≈ 0.2). This likely reflects the inherent complexity of the system, in which distributed and competing active forces act over and are transmitted along a filament with internal degrees of freedom.

To further investigate the relationship between force application and resulting curvature, we calculated the correlation between local force and curvature as a function of the spatial offset ∆x between the force application point and the measured curvature. The correlation between local force and curvature is generally weak, with a peak at ∆x ≈ 0.2 µm (Figure 5h), suggesting that curvature tends to arise slightly displaced with respect to the site of force application. This behavior can be intuitively understood with an analogy to a flexible rod that is exposed to compression: the resulting bend is not maximal at the point of force application but depends on how the rod distributes stress along its length. Soft microtubules show weaker correlations that decay more strongly with distance ∆x compared to stiff microtubules (Figure 5h). This is consistent with their higher susceptibility to noise, which makes their mechanical response less deterministic—also evident in their chaotic looping behavior (Figure 5e, bottom).

Finally, we examined how local motor forces relate to microtubule breakage. In our experiments, microtubules consistently break at regions of high curvature (Figure 4h), similar to what is seen in cells (Figure 2b, bottom; [27]). Our measurements show that the curvature at sites of breakage is three times as high as the mean curvature of buckling microtubules (1.7 µm^−1^ compared to 0.48 µm^−1^, see Figure 4h). Consistent with the low correlation between force and curvature, our simulations reveal that for a given curvature, the force per microtubule segment varies substantially (see Supplementary Figure S6c for a soft microtubule and Supplementary Figure S6d for a stiff microtubule). Nevertheless, a clear trend emerges: higher local curvatures tend to be related to a slightly higher mean force per motor, both in soft and stiff microtubules (Figure 5i). Notably, the model predicts the largest forces to be associated with curvatures in the range of 1 µm^−1^ (for stiff microtubules) to 2 µm^−1^ (for soft microtubules), which coincides with the curvature range where microtubule breakage is observed experimentally (median curvature at breakage is indicated by the dashed line in Figure 5i, also refer Figure 4h).

Interestingly, the force exerted on each segment of the filament displays a nonmonotonic relationship with the local curvature (Figure 5i). At low curvatures, the curvature increases gradually with force. As deformation progresses, the system enters a nonlinear force‐curvature regime characterized by an accelerated increase in curvature [43]. At very high curvatures, however, the force begins to decline, coinciding with topological transitions in the filament, such as the formation of loops. In this regime, the force required for further deformation may decrease, driven by abrupt, large‐scale conformational changes of the filament. Overall, the model reveals that local curvature and local motor‐generated forces only weakly correlate and are not linked in a simple manner.

### 2.6 Microtubule Damage Arises from a Combination of Curvature and Motor Action

**FIGURE 6:**
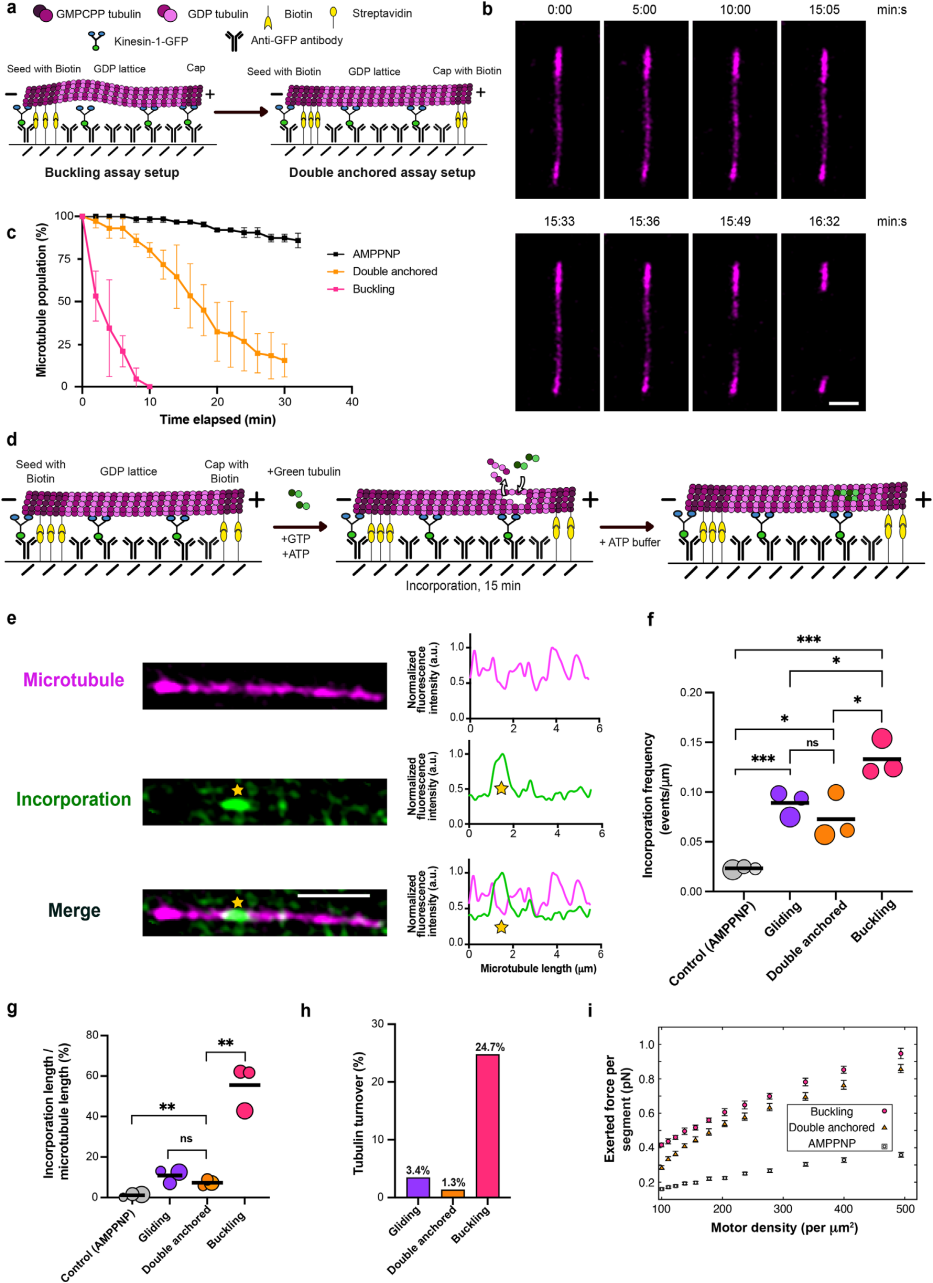
Combination of curvature and motor action contribute to extensive damage in buckling microtubules. (a) Schematic of the experimental setup used to subject microtubules to pulling forces of kinesin (double‐anchored assay). In this assay, both ends of the capped microtubule are anchored to the surface via biotin/streptavidin. (b) Time‐lapse sequence of a double‐anchored microtubule subjected to motor‐induced pulling forces in the absence of free tubulin. Scale bar: 2 µm. (c) Comparison of % microtubule population remaining in control (AMPPNP), double anchored and buckling assays over time in the absence of free tubulin. The symbols indicate mean ± S.D. (n> 100 microtubules analyzed in each condition from two independent experiments) (d) Schematic of the experimental setup used to assess self‐repair in double‐anchored microtubules. (e) Example image showing incorporation of green labeled tubulin (marked with a yellow star) in a microtubule (magenta) in the double‐anchored assay. Scale bar: 2 µm. Graphs represent line scans of the microtubule (magenta) as well as the incorporation channel (green). Profiles have been normalized to 1 for the maximum value of the microtubule and incorporation channel, respectively. (f) Bubble plot showing lower frequency of incorporations in double‐anchored microtubules compared to buckling microtubules. Bubble sizes scale with the total microtubule length analyzed. Each circle represents an independent experiment. Data from three independent experiments comprising 98, 139, and 110 µm of the total microtubule length analyzed for double anchored microtubules. Refer Figure 3g for details on total microtubule length analyzed for gliding and buckling microtubules. Black lines represent the mean. *p =* 0.0242 (for double‐anchored‐buckling); *p* = 0.0212 (double‐anchored ‐control); *p =* 0.3432; not significant (double‐anchored‐gliding) using unpaired *t*‐test. (g) Bubble plot showing a lower percentage of lattice length with incorporation, estimated as incorporation length/ microtubule length, in the double‐anchored assay when compared to buckling as well as control (AMPPNP). Bubble sizes scale with the total microtubule length analyzed. Each circle represents an independent experiment (comprising 98, 139, and 110 µm of total microtubule length analyzed for double anchored microtubules; Refer Figure 3h for details on total microtubule length analyzed for gliding and buckling microtubules). Black lines represent the mean. *p =* 0.0017 (double anchored ‐buckling) and *p =* 0.1415, not significant (double anchored ‐gliding); *p =* 0.0077 (double anchored ‐control‐AMPPNP) using unpaired *t*‐test. Refer Figure 3h for the *p*‐values comparing control (AMPPNP), gliding and buckling. (h) Lesser % of tubulin turnover in double anchored microtubules when compared to buckling microtubules. Total length of microtubules analyzed: 567 µm for gliding, 347 µm for double anchored and 474 µm for buckling microtubules from three independent experiments per condition. Refer to the methods for estimation of % tubulin turnover and Supplementary Figure S7e for estimation of amount of lateral tubulin incorporation. (i) Effect of motor protein arrangement and mobility for three configurations: (1) motors distributed across the surface (leading to buckling), (2) motors arranged linearly beneath a straight microtubule (mimicking double‐anchored microtubules), and (3) in presence of AMPPNP. Total forces exerted on each discretized node of the microtubule are plotted as functions of motor density. Parameters: L = 10 µm and L_p_ = 5 mm. Data are averaged over time and across five microtubules.

To summarize our findings so far, we have established that: (i) Static curvature is sufficient to induce microtubule damage and self‐repair, but it neither leads to microtubule breakage nor reaches the levels of tubulin turnover observed in cells. (ii) Dynamic buckling, in contrast, causes extensive lattice disruption and frequent breakage, which typically occurs in regions of high curvature. (iii) Modeling reveals that local curvature and local motor‐induced force patterns correlate only moderately, yet overall higher forces give rise to higher mean curvatures. Together, these observations indicate that curvature alone cannot account for the severe disruption seen during buckling, and suggest that curvature acts in concert with motor motility [9] and motor‐induced forces to damage microtubules. To experimentally disentangle the contribution of force from curvature, we modified our buckling assay to isolate the effects of force. Specifically, we included biotinylated tubulin in both microtubule ends (seed and cap), allowing microtubules to be anchored at both ends (see **Methods**, Figure 6a). This prevents buckling and keeps microtubules straight but still allows surface‐attached motors to exert pulling forces on the microtubule lattice. We will refer to these microtubules as “double‐anchored microtubules” hereafter.

Figure 6b shows an example of such a double‐anchored microtubule in the absence of free tubulin. Despite remaining straight, the microtubule eventually breaks and disassembles after approx. 16 min (Supplementary Movie S12). A survival analysis (Figure 6c) confirms that double‐anchored microtubules are significantly more stable than buckling ones, though much less so than static controls without active motors.

We reasoned that the reduced damage observed in the absence of free tubulin should result in less tubulin incorporation in the presence of free tubulin. To test this, we added free, green‐labeled tubulin to the assay for 15 min (Figure 6d). Double‐anchored microtubules show occasional incorporation of free tubulin, as seen in the example in Figure 6e. Quantification (Figure 6f–h; Supplementary Figure S7a,e) reveals that incorporation levels are slightly elevated compared to static microtubules, but remain significantly lower than those observed in buckling microtubules. This indicates that double‐anchored microtubules undergo limited damage and self‐repair, consistent with their increased stability in the absence of free tubulin, when compared to buckling microtubules. We did not observe a preferred location of breakage in double‐anchored microtubules (Supplementary Figure S8).

To determine whether these differences could be explained by differing motor forces, we compared the mean motor‐generated force per microtubule segment between double‐anchored and buckling microtubules in simulations across a range of motor densities (Figure 6i; Supplementary Figure S7b,c). The results show that double‐anchored microtubules experience forces of comparable magnitude to buckling microtubules—particularly at intermediate and high motor densities, as used in our experiments—and far greater than in static bent (Supplementary Figure S7d) as well as AMPPNP conditions.

In conclusion, despite being subjected to similar levels of motor‐generated forces, only buckling microtubules exhibit extensive damage and self‐repair. The comparable levels of tubulin loss and incorporation in gliding and double‐anchored microtubules further suggest that motor motility, in addition to motor‐generated forces, contributes to lattice disruption. Thus, lattice damage likely arises from motor movement (and the forces associated with it) and is markedly amplified in zones of high curvature. In summary, our observations support the view that damage in buckling microtubules stems from a combination of curvature and motor action, with force playing a surprisingly minor role and curvature acting as the key amplifier of motor‐induced damage.

### 2.7 Intracellular Factors Enhance Microtubule Resilience to Buckling‐Induced Damage

The extensive damage observed in buckling microtubules, which often surpasses their intrinsic self‐repair capacity, prompted us to explore how microtubules withstand mechanical stress in the intracellular environment—where bending and buckling are common, as in our in vitro system. Several microtubule‐associated proteins (MAPs) have been implicated in promoting microtubule resilience to mechanical stress [13, 44, 45, 46]. We therefore hypothesized that intracellular factors may protect microtubules from damage and help maintain their structural integrity.

**FIGURE 7:**
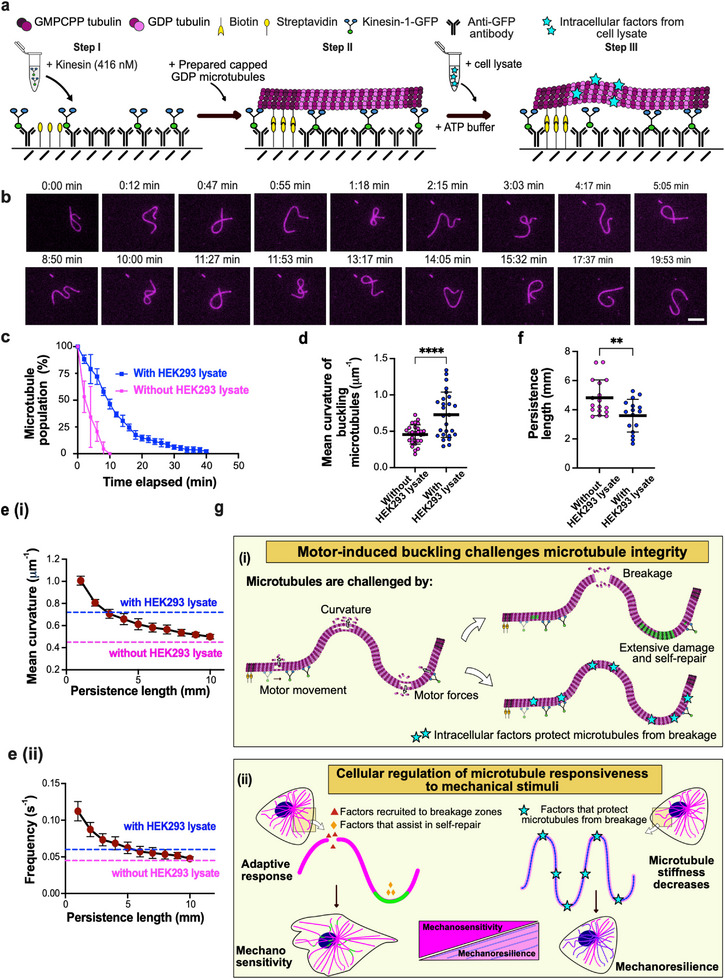
Intracellular factors protect buckling microtubules from breakage. (a) Schematic of the experimental setup used to assess the influence of intracellular factors on microtubule survival in buckling assays: In step I, similar to the gliding and buckling assay setups, purified kinesin‐1‐GFP (416 nM) is immobilized on to an anti‐GFP antibody coated surface. Then, prepared capped microtubules (step II) are added. Microtubule buckling (step III) is achieved by flushing in a mix containing ATP buffer (GTP, ATP) with 20 µg mL^−1^ HEK293 cell lysate. (b) Time‐lapse sequence showing survival of a buckling microtubule in presence of 20 µg mL^−1^ HEK293 cell lysate. Scale bar: 5 µm. (c) Buckling microtubules survive longer in presence of cell lysate. Comparison of the percentage of the microtubule population remaining (in the absence of free tubulin) over time in the case of buckling microtubules both with and without 20 µg mL^−1^ HEK293 lysate (n > 100 microtubules analyzed in each condition from two independent experiments). The symbols and error bars indicate mean ± S.D, respectively. (d) Comparison of mean curvature in buckling microtubules in the presence and absence of HEK293 cell lysate. Black line represents the mean and error bars represent the S.D. p < 0.0001 using an unpaired *t*‐test. (Mean obtained from analyzing microtubule mean curvature at different timepoints from 4 microtubules in each case: n = 28 for without HEK293 lysate condition and n = 26 for with HEK293 lysate condition. Refer Supplementary Figure S9g for comparison of lengths of microtubules analyzed). (e, i) Mean curvature and (ii) oscillation frequency (bottom) as functions of microtubule persistence length at L = 10 µm and ρ = 400 µm^−2^. The symbols and error bars indicate mean ± S.D, respectively. Blue and pink dotted lines represent experimentally determined values with and without HEK293 lysate, respectively. (f) Microtubules are 1.35‐fold softer in the presence of cell lysate. Plot showing comparison of persistence length (in mm) of microtubules with and without 20 µg mL^−1^ HEK293 cell lysate estimated by thermal fluctuation experiments (refer Methods). Black line represents the mean and error bars represent the S.D. *p* = 0.0054 using an unpaired *t*‐test. Persistence length was estimated from analyzing atleast 60 frames for each microtubule over 20 mins for both conditions. n = 17 microtubules for without HEK293 lysate condition and n = 16 for with HEK293 lysate condition from three independent experiments each. (g, i) Buckling microtubules are challenged by the combination of motor motility, forces and curvature resulting in extensive damage and self‐repair or breakage. Intracellular factors protect microtubules and help enhance their survival under sustained deformation. (g, ii) Our findings suggest that cells may use intracellular factors to regulate microtubule response to mechanical stimuli by factors specifically recruited to microtubule breakage zones and repair sites (mechanosensitivity) and by factors that decrease microtubule stiffness (mechanoresilience).

To test this idea, we examined the survival of buckling microtubules in the presence of cell lysate from HEK293 cells, but in the absence of free tubulin (Figure 7a). While the exact composition of the lysate is unknown, it contains soluble cytoplasmic components and could thus influence microtubule stability. Because cell lysate has previously been shown to modulate motor activity [47], we used a low concentration of 60 µg mL^−1^ or below to minimize such effects, as shown in Korten et al., 2013 [48]. Using low concentrations of cell lysate also ensures that any residual tubulin present is negligible. Tubulin constitutes 2%–3.5% of the total cellular protein content [49, 50], and in our assay conditions the corresponding tubulin concentration falls within the picomolar range (Supplementary Figure S9a–c), rendering it unlikely to contribute measurably to microtubule repair. We tested the activity of kinesin motors both in the absence and in the presence of lysate and found the microtubule gliding velocity to be similar Supplementary Figure S9d). Examples of buckling microtubules in the presence of 20 µg mL^−1^ HEK293 cell lysate and 60 µg mL^−1^ PtK2 cell lysate are shown in Figure 7b and Supplementary Figure S9e, respectively.

We observed that the addition of lysate during buckling increases microtubule survival: buckling microtubules remain intact for up to ∼40 min, significantly longer than without lysate (Figure 7c, Supplementary Figure S9f). Since only negligible amounts of free tubulin are present in cell lysates, this enhanced survival suggests that factors present in the lysate reduce tubulin loss and stabilize the microtubule lattice. Interestingly, when comparing buckling microtubules in the absence and presence of lysate (Figures 2d vs. 7b; Supplementary Movie S13), we noticed that microtubules visually appear softer in the lysate condition, since they buckle faster and with more pronounced curvature. This qualitative impression is supported by curvature quantification, which shows higher mean curvatures in the presence of lysate (Figure 7d, Supplementary Figure S9e). To better understand these observations, we used our computational model to compare the buckling behavior of soft (L_p_ = 1 mm) and stiff (L_p_ = 10 mm) microtubules. Soft microtubules show both a higher buckling frequency and greater mean curvature (Supplementary Figure S10a), resembling the experimental data in the presence of lysate. In simulations, this experimental difference can be recapitulated by an approximate twofold reduction in the persistence length (Figure 7e(i,ii)). Consistent with these predictions, estimates of microtubule persistence length derived from thermal fluctuation analysis (see Methods) reveals a 1.35‐fold decrease in persistence length in the presence of 20 µg mL^−1^ HEK293 cell lysate (Figure 7f).

Alternatively, intracellular factors could modulate motor motility parameters. Model predictions indicate that reproducing the lysate data would require either a two to sevenfold increase in the attachment rate ω_a_ (Supplementary Figure S10b) or a roughly twofold decrease in the detachment rate ω_d_ (Supplementary Figure S10c). Importantly, both scenarios increase the effective number of motors attached and thus cannot explain the higher survival in lysate, which would require the opposite effect (fewer attached motors). We therefore consider changes in motor parameters alone unlikely to account for the experimental data, unless additional protective factors overcompensate the increased damage.

Although we cannot currently identify the molecular players responsible for this effect, our findings are consistent with the possibility that intracellular factors contribute to microtubule survival under mechanical stress by enhancing microtubule flexibility. Increased flexibility may allow microtubules to adopt strongly bent conformations without breaking, thus preserving lattice integrity under sustained deformation.

## 3 Discussion

In this study, we highlight the limits of intrinsic microtubule self‐repair under mechanical stress induced by molecular motors and show that additional intracellular factors are required to maintain microtubule integrity during sustained dynamic buckling. Using an in vitro assay that mimics kinesin‐driven buckling as observed in cells, we find that the combination of motor motility, motor‐induced forces and high curvature inflicts substantial lattice damage that often exceeds microtubule self‐repair capacity.

Previous work has established that kinesin motility itself can induce lattice damage and trigger tubulin incorporation along the lattice, even when the motors are not under load [9, 10, 11]. In these contexts, however, lattice damage is typically limited and largely counteracted by intrinsic self‐repair, allowing microtubules to maintain structural integrity. Our experiments extend these findings by demonstrating that when kinesin activity is coupled to dynamic buckling, microtubule damage is induced by the combined effects of motor motility, active forces, and microtubule curvature. While forces of ∼30 pN applied in motor‐independent assays have been shown to extract tubulin dimers from the lattice, and coupled kinesin motors can likewise disrupt the lattice [51], our double‐anchored assays display levels of microtubule damage and self‐repair comparable to gliding microtubules and markedly lower than those observed under buckling conditions. This suggests that motor generated forces contribute to a similar extent as motor motility. By disentangling curvature, motor‐driven gliding, and motor generated force using static bending, gliding, and double anchored configurations, respectively, we show that each factor alone produces comparatively modest lattice damage. In contrast, their simultaneous action during buckling causes extensive damage that often exceeds intrinsic self‐repair capacity, leading to localized breakage at regions of high curvature. These observations suggest that curvature acts as a critical amplifier of motor‐induced lattice damage, thereby defining a mechanical regime in which previously described self‐repair mechanisms become insufficient. Consistently, a recent study reported enhanced bending, buckling, and microtubule fragmentation in cells containing kinesin condensates formed upon overexpression of kinesin‐3 [52], in line with our observation of catastrophic filament failure at high motor densities.

Among the cytoskeletal filament types, microtubules stand out due to their unusual combination of properties: they are relatively stiff polymers, yet remarkably responsive to biochemical and mechanical stimuli [3, 14, 18]. Since the discovery of microtubule self‐repair, it has been widely assumed that this mechanism alone is sufficient to counteract the mechanical forces and damage they sustain in cells [6, 9, 14, 15]. However, our results challenge this notion. They reveal that in situations where microtubules are subjected to dynamic buckling by continuous motor activity, leading to high curvatures, as seen in cells, intrinsic self‐repair mechanisms can be overwhelmed. In contrast, the presence of intracellular components markedly enhances microtubule resilience, enabling microtubules to survive under mechanically demanding conditions (Figure 7g(i)).

Interestingly, microtubules show higher curvatures and are 1.35‐fold times softer in the presence of cell lysate than in buffer alone, suggesting that intracellular factors may potentially contribute to microtubule resilience by modulating their mechanical properties. While changes in motor parameters such as altered attachment or detachment rates could also contribute to the observed buckling behavior, such changes alone would not readily account for the increased survival of microtubules in the presence of cell lysate. This observation therefore raises the possibility that microtubule stiffness in cells may be lower than previously estimated from in vitro measurements [18, 53, 54]. Reduced stiffness could serve as a protective function by allowing microtubules to adjust their shape under load—a form of mechanical compliance that may potentially be actively regulated in cells. For example, cells may selectively increase microtubule flexibility through recruitment of MAPs that reduce microtubule rigidity [55, 56]. Tubulin post‐translational modifications, particularly acetylation, have been proposed to enhance microtubule flexibility by weakening the lateral interactions between tubulin dimers [57]. However, in our experiments, it is unlikely that acetylating enzymes (if any) from the cell lysate contribute significantly to the observed microtubule resilience, as microtubules are subjected to mechanical stress from the onset of the experiment, whereas tubulin acetylation is characterized by comparatively slow enzymatic kinetics (catalytic rate of the acetylating enzyme αTAT1 is 0.4 h^−1^) that are unlikely to counteract early damage events [58].

More broadly, our findings suggest the intriguing possibility that cells may fine‐tune microtubule rigidity and responsiveness to mechanical signals depending on functional needs (Figure 7g(ii)). For example, primary cilia, which serve as sensory organelles, may benefit from the ability of microtubules to respond to subtle mechanical stimuli (mechanosensitivity), whereas motile cilia may require less sensitive microtubules to withstand repetitive mechanical stress (mechanoresilience). In plant cells, highly organized microtubule arrays guide morphogenesis by aligning with tensile stress patterns, often forcing them into strongly curved conformations [59]. By differentially regulating stiffness and stress responsiveness, cells may thus navigate the conflict between cell geometry and tension patterns. A better understanding of how microtubule properties are regulated in different contexts could reveal cell‐type specific adaptations and vulnerabilities.

## 4 Materials and Methods

### 4.1 Cell Culture and Live Cell Imaging

Male potoroo kidney epithelial cells (PtK2), stably expressing GFP‐Tubulin (displayed in Figure 2 in Magenta) were cultured at 37°C and 5% CO_2_ in DMEM/F12 media (31331028, Gibco), supplemented with 10% Fetal Bovine Serum (FBS, Gibco A5670701) and 1% Penicillin‐Streptomycin solution (15070063, Gibco). Human Embryonic Kidney cells (HEK293, DMSZ, ACC305) were cultured in Complete DMEM (high glucose with HEPES) media (Fisher Scientific, 42430025) with 10% Fetal Bovine Serum (heat inactivated at 56°C) and 1% Penicillin‐Streptomycin solution at 37°C and 5% CO_2_. For live cell imaging, PtK2 cells were seeded in confocal glass‐bottom dishes (734‐2904, VWR Avantor) coated with 0.01 mg mL^−1^ fibronectin, a day prior to imaging. Live cell imaging for observing dynamic microtubules was carried out using a 63× immersion oil objective (Zeiss) maintained at 37°C and 5% CO_2_. Time‐lapses of bending and buckling events in PtK2 cells were captured for a period of 5 min.

### 4.2 Purification and Labelling of Tubulin

Tubulin free of Microtubule Associated Proteins (MAPs) was purified from fresh calf brains by three cycles of polymerization‐depolymerization, using a combination of low and high salt buffers, followed by cation exchange chromatography, as previously described in [60]. Briefly, the first polymerization‐depolymerization cycle was performed in low salt conditions (0.1 M PIPES, 0.5 mM MgCl_2,_ 2 mM EGTA and 0.1 mM EDTA). This was followed by the second cycle in high salt buffer [High Molarity PIPES Buffer: 1 M PIPES, pH 6.9, supplemented with KOH, 10 mM MgCl_2_, 20 mM EGTA]. Subsequently MAP free tubulin was obtained after cation‐exchange chromatography (Fractogel EMD SO3, Merck) in 50 mM PIPES, pH 6.8, supplemented with 1 mM MgCl_2_ and 1 mM EGTA.

Fluorescently labeled tubulin (ATTO‐488 and ATTO‐565‐labeled tubulin) and biotinylated tubulin were prepared according to the protocol described in Hyman et al.,1991 [60]. MAP‐free tubulin obtained after cation exchange chromatography was polymerized at 37°C for 1 h in BRB80 [Brinkley Buffer 80: 80 mM PIPES, pH 6.8, 1 mM EGTA and 1 mM MgCl_2_] supplemented with 33% glycerol, 4 mM MgCl_2_ and 1 mM GTP. The polymerized microtubules were layered onto prewarmed cushions of 0.1 M Na‐HEPES, pH 6.8, 1 mM MgCl_2_, 1 mM EGTA, 60% v/v glycerol. This was followed by high‐speed centrifugation at 37°C for 1 h. Then the pellet was resuspended in 0.1 M Na‐HEPES, pH 8.6, 1 mM MgCl_2_, 1 mM EGTA, 40% v/v glycerol. To this, 1/10^th^ volume of 100 mM NHS‐ATTO‐565/488 (ATTO‐Tec), or NHS‐LC‐LC‐biotin (EZ‐link, ThermoFisher) was added, and the mix was allowed to incubate for 10 min at 37°C. Subsequently, the labeling reaction was stopped using two volumes of BRB80, with 100 mM potassium glutamate and 40% v/v glycerol. The labeled microtubules were sedimented onto BRB cushions with 60% glycerol. Following this, an additional cycle of polymerization and depolymerization was performed before the labeled tubulin was aliquoted, snap‐frozen and stored in liquid nitrogen till use.

### 4.3 Purification of Kinesin‐1‐GFP Motor Proteins

Recombinant, truncated kinesin‐1 motor protein was purified as previously described in [61]. The plasmid encoding for the kinesin‐1 protein (human KIF5B heavy chain truncated to 560 aa; GFP and His tag at the C‐terminus of the insert and backbone, respectively), pET17_K560_GFP_His, was purchased from Addgene (15219, Cambridge, MA). The plasmid was transfected into Rosetta2 (DE3)‐pLysS *E. coli* (VWR) and incubated with 0.2 mM IPTG at 16°C for 16 h. Following this, the cells were resuspended in cation‐exchange buffer (6.67 mM Na‐Acetate, 6.67 mM MES, 6.67 mM HEPES, 20 mM β‐mercaptoethanol (BME), 0.2 mM ATP, and 0.2% Tween‐20, pH 7.0 supplemented with a protease inhibitor cocktail) and lysed by sonication. The lysates were centrifuged at 38 000 × g for 30 min at 4°C and loaded into a HiTrap SP Cation exchange column (HiTrap SP HP, 17‐1151‐01, Cytiva). The column was then washed with cation exchange buffer supplemented with 50 mM KCl, followed by elution with cation exchange buffer supplemented with 300 mM KCl. The eluted fraction was then loaded on to a Ni‐NTA column (HisTrap HPTM, 17‐5247‐01, Cytiva) after dilution with nickel loading buffer (Nickel buffer: 50 mM sodium phosphate buffer, pH 7.5, 5% w/v glycerol, 300 mM KCl, 1 mM MgCl_2_, 0.2% w/v Tween‐20, 10 mM BME, 0.1 mM ATP, supplemented with imidazole to a final concentration of 36 mM). This was followed by washing with nickel washing buffer (nickel buffer supplemented with KCl to a final concentration of 1000 mM and imidazole to a final concentration of 30 mM) and elution with nickel elution buffer (nickel buffer supplemented with imidazole to a final concentration of 300 mM). The collected fractions were centrifuged at 4000 g for 30 min at 4°C. To remove imidazole, the sample was dialyzed overnight against K560 buffer (50 mM Na‐phosphate pH 7.5, 300 mM KCl, 5% glycerol, 1 mM MgCl_2_, 1 mM DTT, 0.1 mM ATP). The sample was then loaded onto a gel filtration Superdex column (Superdex 200 Increase 10/300 GL, 28‐9909‐44, Cytiva) and eluted with K560 buffer. The eluted fractions were concentrated using a 30 kDa membrane filter by centrifugation at 4000×g for 30 min at 4°C. Finally, the protein was snap‐frozen in 5 µL aliquots and stored in liquid nitrogen till use. In the text and in figures, Kinesin‐1‐GFP is represented in cerulean blue (False color LUT).

### 4.4 Preparation of HEK293 Cell Lysates

0.6 million HEK293 cells or PtK2 cells/well were seeded into two 6‐well plates. After 48 h, cell lysates were prepared according to the protocol mentioned in [62]. Briefly, transfected cells were treated with trypsin and centrifuged at 450 × g for 10 min at 4°C. All steps after centrifugation were carried out on ice or at 4°C. The cell pellet (from two 6 well plates) was resuspended in 130 µL ice‐cold lysis buffer ((BRB80 containing 0.05% Triton X‐100 and protease inhibitors (10 µg mL^−1^ leupeptin, aprotinin and 4‐(2‐aminoethyl)‐benzenesulfonyl fluoride; Sigma‐Aldrich)). The resuspended mixture was transferred to an ice‐cold 1.5 mL Beckman ultracentrifuge tube, and the cells were further lysed by pipetting. This was followed by sonication (4 short pulses at 12% Amplitude, MS‐72 probe, Bandelin Sonoplus). The lysed mixture was further mixed by pipetting and then centrifuged at 33 800 × g for 30 min at 4°C. The supernatant was aliquoted, snap‐frozen in liquid nitrogen and stored at −80°C. The total protein concentration was estimated using the Bichinchoninic acid protein assay (Pierce BCA protein assay kit, 23200, ThermoScientific). For buckling assays, 20 µg mL^−1^ of HEK293 and 60 µg mL^−1^ PtK2 cell lysate (final total protein concentration) was added along with ATP buffer.

### 4.5 Western Blot for Estimation of Tubulin Concentration in Cell Lysates

The concentration of tubulin present in cell lysates (both HEK293 and PtK2) was quantified using western blot analysis. A western blot of dilution series of purified tubulin of known concentration as standard and dilution of cell lysates was performed (Refer Supplementary Figure S9a). The samples were boiled for 5 mins at 95°C in 1 × Lamelli buffer (Bio‐Rad), run on a 10% SDS‐PAGE gel and transferred to a nitrocellulose membrane. The transfer was performed in 1 × Blotting buffer (14.4 g Glycine, 3 g Tris‐base, 10% Ethanol) for 1.5 h. The immunoblots were subsequently blocked using 5% non‐fat milk (68514‐61‐4, Roth) in 1 × TBS (Tris‐Buffered Saline). The blots were then incubated overnight in a 1:1000 dilution of alpha tubulin mouse monoclonal antibody (DM1A, 62204, ThermoFisher) in 5% non‐fat milk in 1 × TBS. The immunoblots were then washed thrice with 1 × TBS‐T and incubated in a 1:15 000 dilution of secondary antibody (IRDye680RD goat anti‐mouse antibody, LICORbio, 926‐68070, in 5% non‐fat milk in 1 × TBS) for 1 h. Following this, the blots were washed thrice in 1 × TBS‐T (Tris‐Buffered Saline with 0.1% Tween‐20) before imaging using ChemiDoc gel imager (12009077, Bio‐Rad).

### 4.6 Preparation of GMPCPP Stabilized Microtubule Seeds

Microtubule seeds were prepared by polymerizing 10 µM of tubulin (40% red fluorescent tubulin and 60% unlabeled or biotinylated tubulin) in BRB80 buffer supplemented with 0.5 mM GMPCPP (Guanosine‐5’‐[(α,β)‐methyleno] triphosphate: slowly hydrolysable GTP analogue) (NU‐405, Jena Bioscience) for 1 h at 37°C. Following this, the above mixture was incubated with 1 µM Paclitaxel (Sigma) for 30 min at room temperature, centrifuged (21 300 × g at 25°C for 15 min) and the pellet was resuspended in warm BRB80 supplemented with 0.5 mM GMPCPP. The prepared microtubule seeds were stored in liquid nitrogen until use.

### 4.7 Preparation of Capped GDP Microtubules for In Vitro Assays

Capped GDP microtubules in a single color (displayed in figures in magenta) were prepared with a lower proportion of fluorescent tubulin (12%) in the GDP lattice in contrast to the stabilized ends (40% labeled seeds and caps). Capped GDP microtubules were prepared by elongating prepared microtubules seeds with 10 µM of tubulin (12% labeled) in Elongation buffer (56 mM PIPES, 0.7 mM EGTA, 0.7 mM Mgcl_2_, 38 mM KCl, 19 mM Phosphate buffer, pH 6.8), supplemented with 1 mM GTP for 40 min at 37°C. The sample with the polymerized microtubules was then immediately centrifuged (21 300 × g for 15 min). The resulting pellet was resuspended in a resuspending mix containing Elongation Buffer supplemented with 0.5 mM GMPCPP and further incubated at 37°C to cap the ends of the microtubule. Capping helps prolong the lifetime of the microtubule and additionally allows for better visualization of lattice self‐repair. The resuspended mix is then successively capped by adding 0.5 µM of tubulin (60% biotin, 40% fluorescent tubulin) in a stepwise manner (for a total of 10 times) every 15 min.

For microtubule static curvature and double anchored motor assays, GDP microtubules with seeds and caps containing biotin were prepared. For microtubules buckling assays, GDP microtubules were prepared using biotin‐containing seeds and the caps were grown using 0.5 µM (60% unlabeled and 40% fluorescent) tubulin. For microtubules gliding assays, GDP microtubules were prepared using seeds (60% unlabeled and 40% fluorescent, without biotin) and the caps were grown using 0.5 µM tubulin (60% unlabeled and 40% fluorescent).

### 4.8 Cover‐Glass Passivation

For experiments analyzing static curvature of microtubules, cover‐glasses were passivated with Silane‐PEG‐Biotin as follows: Cover‐glasses were first wiped with lint‐free KimWipe (Kimberly‐Clark Professional 33670‐04) tissues and 70% ethanol, incubated in Acetone for 30 min, followed by 96% ethanol for 15 min (gentle agitation at room temperature). The cover‐glasses were subsequently rinsed in ultrapure water, incubated in Hellmanex III solution (2% in water, Hellmanex) for 2 h (gentle agitation at room temperature), washed in ultrapure water and dried. This was followed by treatment using an Deep‐UVO cleaner (30 mW/cm^2^ at 254 nm, 144AX‐220 Jelight) for 30 min before incubation in 7:3 mix of tri‐ethoxy‐silane‐PEG and tri‐ethoxy‐silane‐PEG‐biotin (30 kDa, Creative PEG works) (final concentration of 1 mg mL^−1^ in 96% ethanol and 0.1% HCl), for 3 days with gentle agitation at room temperature. After 3 days, the PEGylated cover‐glasses were extensively rinsed in 96% ethanol and ultrapure water, air‐dried and stored at 4°C till use.

For gliding and buckling assays with kinesin‐1 motor proteins, cover‐glasses were wiped with lint‐free KimWipe tissues and 96% ethanol, rinsed in ultrapure water and sonicated in 2% Hellmanex‐III solution at 60°C for 30 min. Following sonication, the cover‐glasses were rinsed in ultrapure water, stored in ultrapure water at room temperature and air‐dried just before use.

### 4.9 Assay to Assess Self‐Repair in Static Bent Microtubules

A flow chamber of an approximate volume of 60 µL was built by sandwiching two pieces of SiPEG‐Biotin passivated cover‐glasses using two strips of double‐sided adhesive tape (70 µm height, 0000P70PC3003, LiMA, Couzeix, France). The flow chamber was first perfused with streptavidin (100 µg mL^−1^ in 1 × BRB80; Fisher Scientific) for 1 min. This was followed by a solution of 0.1 µg mL^−1^ PLL‐g‐PEG (Pll 20 K‐G35‐PEG2K, Jenkam Technology, in 10 mM Na‐HEPES, pH 7.4) for 1 min. Following a wash step with 1 × BRB80, prepared capped GDP microtubules (diluted in 1 × BRB80) were flushed in an alternative manner (to achieve bending) and incubated for 5 min before extensive washing with BRB80 supplemented with 1 mg mL^−1^ BSA (Bovine Serum Albumin, Sigma). Subsequently, an incorporation mix containing 5 µM Tubulin (100% ATTO‐488, green labeled) in Elongation buffer supplemented with 1 mM GTP, an oxygen scavenger cocktail (22 mM DTT, 1.2 mg mL^−1^ glucose, 8 µg mL^−1^ catalase and 40 µg mL^−1^ glucose oxidase), 1 mg mL^−1^ BSA and 0.033% (w/v) methyl cellulose (1500 cP, Sigma) was added and allowed to incubate at 37°C for 15 min. Owing to the high background resulting from usage of 100% labeled tubulin, incorporation events can only be detected after washout of free tubulin, prior to imaging. Therefore, following incubation, the chamber was perfused with 200 µL of imaging buffer (composition same as that of the incorporation buffer with an addition of 2 µM unlabeled tubulin‐to keep microtubules stable for imaging). The chamber was then sealed with Valap before imaging.

### 4.10 Microtubule Gliding Assay

In vitro gliding assays were performed in 20 µL flow chambers constructed from Hellmanex‐sonicated cover‐glasses and using double‐sided tape. The chamber was first perfused with a 20 µL solution of 0.2 mg mL^−1^ Anti‐Green‐Fluorescent‐Protein (GFP) Antibody (Invitrogen, A‐11122) for 3 min. This was followed by further passivation using 1% w/v BSA solution in 1×HKEM buffer (10 mM HEPES buffer (pH 7.2), 50 mM KCl, 1 mM EGTA and 5 mM MgCl_2_). Kinesin‐1‐GFP diluted in TicTac buffer (10 mM HEPES buffer (pH 7.2), 16 mM PIPES buffer (pH 6.8), 50 mM KCl, 5 mM MgCl_2_, 1 mM EGTA, 20 mM dithiothreitol (DTT), 3 mg mL^−1^ glucose, 20 µg mL^−1^ catalase, 100 µg mL^−1^ glucose oxidase and 0.3% BSA) was added and allowed to incubate for 5 min. Subsequently the chamber was washed with TicTac buffer and prepared capped GDP microtubules (diluted in 1×BRB80) was added and allowed to incubate for 2 min, followed by extensive washing with TicTac buffer. ATP Buffer (10 mM HEPES buffer (pH 7.2), 56 mM PIPES buffer (pH 6.8), 50 mM KCl, 5 mM MgCl_2_, 1 mM EGTA, 20 mM dithiothreitol (DTT), 3 mg mL^−1^ glucose, 20 µg mL^−1^ catalase, 100 µg mL^−1^ glucose oxidase, 0.3% BSA supplemented with 2.7 mM of ATP and 0.2% methyl cellulose) was then added to initiate motor activity. The chamber was sealed with Valap and immediately imaged. For control experiments, ATP was replaced with 2.7 mM of AMPPNP (Adenosine‐5′‐(β,γ‐imido) triphosphate; A2647, Sigma‐Aldrich) in the final buffer mix. For experiments with survival in the presence of free tubulin, 5 µM tubulin (100% unlabeled) was added to the ATP buffer. For gliding assays in the presence of HEK293 cell lysate, 20 µg mL^−1^ of cell lysate was added in the ATP buffer (all other steps were kept the same).

### 4.11 Microtubule Buckling Assay

To achieve microtubule buckling, the flow chamber was first perfused with 100 µg mL^−1^ of streptavidin (diluted in 1 × HKEM, Invitrogen, 434301) prior to the anti‐GFP‐antibody step. Capped GDP microtubules made from biotinylated seeds were used (all other steps were kept the same). All other steps were the same as the microtubule gliding assay described above. For buckling assays in the presence of HEK293 cell lysate, 20 µg mL^−1^ of cell lysate was added in the ATP buffer.

### 4.12 Incorporation in Microtubule Gliding and Buckling Assays

To visualize self‐repair (incorporation of free tubulin dimers) in buckling and gliding microtubules, an additional coating step (TicTac buffer supplemented with 2 µM unlabeled tubulin) was performed prior to addition of prepared GDP microtubules as free green tubulin dimers tend to attach to the layer of motors during the assay, effectively hindering the detection of the incorporated dimers [9]. To visualize self‐repair, gliding and buckling microtubules were exposed to ATP buffer supplemented with 5 µM tubulin (100% labeled) and allowed to glide/buckle at 37°C for 15 min. Following incubation, the chamber was perfused with ATP buffer containing 2 µM unlabeled tubulin (to stabilize microtubules for imaging), sealed with Valap before imaging.

### 4.13 Single Molecule Photobleaching Experiments to Determine Kinesin Surface Density

Single molecule photobleaching (SMPB) was performed according to the protocol described previously in [63]. Briefly, a 1 µM dilution of Kinesin‐1‐ GFP in cold 1×HKEM was centrifuged to remove aggregates (15 min, 4°C, 215 000 × g in a Type 70 Ti rotor [Beckman Optima XPN80]) prior to the assay. In a 20 µL flow chamber made from Hellmanex sonicated cover‐glasses, 20 µL of 0.2 mg mL^−1^ solution of Anti‐GFP antibody was flushed in and incubated for 3 min. This was followed by a 1 × HKEM wash step and then a 350 pM solution of Kinesin‐1‐GFP (further diluted in 1 × HKEM) was added and incubated for 5 min. The chamber was then washed with 300 µl of 1 × HKEM to remove unbound Kinesin‐1 molecules and sealed using Valap. Photobleaching was achieved at 130 mW laser power with 500 ms exposure time (for 5 min) in continuous streaming mode using a 100× Olympus objective on an orbital TIRF Nikon Ti2e microscope. Recorded time‐lapses were cropped and regions of interest (ROIs) with uniform illumination were chosen for analysis. Using Stowers Institute Fiji plugin, the fluorescent intensity traces of individual Kinesin‐1‐GFP molecules (represented in cerulean blue in Supplementary Figure S5a(i)) were obtained. From the step‐like traces (refer Supplementary Figure S5a(ii)), we quantified the average fluorescent intensity of one Kinesin‐1 molecule. To quantify the surface density of Kinesin‐1 (refer Supplementary Figure S5b) at our working concentration of 416 nM, we repeated the above assay with 416 nM of kinesin‐1‐GFP and captured images using the same imaging conditions (65% Laser power with 500 ms exposure time, same camera gain and binning settings as above). ROIs with uniform illumination were chosen for analysis and average intensity of fluorescent Kinesin‐1 at 416 nM was estimated. The experiment was performed on the same day and using the same imaging settings as the SMPB assay. The surface density (No: of Kinesins/µm^2^) was estimated by dividing the average intensity of 416 nM Kinesin/ [(Average intensity of one Kinesin‐1 molecule) * (Pixel size)^2^].

### 4.14 Single‐Molecule Motility Experiments to Estimate Kinesin Motility Parameters

For single molecule experiments to estimate motility parameters of Kinesin‐1‐GFP molecules (represented in cerulean blue in Supplementary Figure S5), an orbital TIRF microscope was used. Prior to the assay, a 1 µM dilution of Kinesin‐1‐GFP in cold 1 × HKEM was centrifuged to remove aggregates (15 min, 4°C, 215 000 × g). In a 20 µL flow chamber made from PEGylated cover‐glasses, 20 µL of streptavidin (50 µg mL^−1^ in 1 × HKEM; Fisher Scientific) was flushed in and allowed to incubate for 1 min. Following a wash step with 1 × HKEM, prepared capped GDP microtubules with biotinylated seeds and caps (diluted in 1 × BRB80) were flushed in and incubated for 5 min before extensive washing with 1 × HKEM. Subsequently, motility buffer containing 500 pM Kinesin‐1‐GFP in TicTac buffer (containing the oxygen scavenger solutions to minimize bleaching of fluorophores) was flushed in. One still image of the microtubule was acquired. Motile kinesin‐1 single molecules were captured in continuous streaming mode in the GFP channel (using the same image conditions as in the SMPB assay). The time‐lapses were processed in Fiji and kymographs (Refer Supplementary Figure S5c) were generated using the KymoResliceWide plugin. Tracking the traces of kinesin‐1‐GFP molecules from kymographs from 50 frames with a temporal cutoff of 500 ms, distributions of the run length (distance traveled by an individual kinesin‐1 molecule on a microtubule), dwell time (total residence time of individual kinesin‐1 molecule on a microtubule), mean velocity and detachment rate of kinesin‐1 molecules were estimated (Supplementary Figure S5d–g).

### 4.15 Imaging

63× oil immersion objective (Zeiss, Plan‐Apochromat 63×/ NA = 1.40 oil DIC M27) of a Zeiss LSM 900 confocal microscope with an Axiocam 705 Mono camera (Zeiss) and Laser module 5 URGB (with laser lines 405, 488, 561, and 640 nm) was used for imaging. The microscope stage was kept at 37°C by means of a warm stage controller (Insert‐P, PeCon). The temperature on the microscope stage was controlled with the incubator (PeCon) kept at 37°C. Time‐lapses were recorded using ZenBlue software (version 3.2, Zeiss). Images were acquired of simultaneously of both 488 (using 0.08 mW laser power) and 565 (using 0.12 mW laser power) channels with a frame interval of 600 ms. Single‐molecule experiments were performed on an objective‐based orbital TIRF microscope (Nikon Ti2 Eclipse, modified by ViSitron Systems) equipped with an EMCCD Camera (Andor iXon Life) and Visitron Orbital 600 module with VS Laser Merge system with 4 laser lines (405, 488, 561, and 640). A 100× Olympus UPlanApo TIRF objective (100×/1.5 oil, correction collar set to 0.17 mm to match cover‐glass thickness) was used for single‐molecule experiments. Images were acquired of the 488‐channel using 130 mW laser power and 500 ms exposure in continuous streaming mode for both photobleaching and single molecule motility experiments. The microscope stage was maintained at 37°C using a warm stage controller (OkoLabs). Time‐lapses were recorded using VisiView software (version 6.0). Microtubule thermal fluctuations time‐lapses were imaged using the 60× Oil immersion objective (NA = 1.42; correction collar set to 0.17 mm to match cover‐glass thickness) of a Nikon Ti2E Epifluorescence microscope equipped with Lumencor Spectra III light engine, with solid ‐state illumination (380–750 nm). Images were acquired of the 565‐LED using 10 mW laser power, 300 ms exposure with a frame interval of 10 s. Time lapses were recorded using NIS elements AR software (Nikon).

### 4.16 Image Processing and Quantification of Incorporation Stretches

Videos were processed to improve the signal/noise ratio (subtract background and smooth functions of Fiji, version 1.53t) [64]. Self‐repair events or incorporations were estimated from overlaid images (average of 3 frames) from time‐lapses taken every 2 s. Incorporation stretches were identified from line scans (green fluorescence) along the magenta GDP microtubule lattice. A stretch of green fluorescence along the microtubule (in magenta as indicated in Figure 1c) was regarded as an incorporation if it displayed a fluorescence intensity higher than 1.5 times that of the background as well as followed the lateral fluctuations of the microtubule. A decrease in the normalized intensity of the microtubule lattice is often observed at incorporation sites indicating self‐repair (See Supplementary Figure S4). The full‐width‐half‐maximum (FWHM) distance from the intensity profile of the incorporation stretch was taken as the incorporation length. Accounting for the resolution limit of the microscope, incorporation stretches spanning less than 250 nm were disregarded from analysis.

For data on microtubule self‐repair in cells, images of a microinjected cell were first divided into 4–5 ROIs (regions of interest, as indicated in Figure 1g with a box with a red outline) and sections of microtubules (indicated with boxes with white outline in Figure 1g) were analyzed. Incorporations found on bundled microtubules and at microtubule crossover sites (and up to 0.8 µm away from crossover sites) were disregarded from analysis. As mentioned in [29], longer incorporation stretches were found in microtubules located close to the microinjection sites owing to possible damage from microinjection. Hence, only microtubule sections found 10 µm or more from the microinjection site were considered for analysis.

### 4.17 Curvature Analysis

Images of both straight and bent microtubules were tracked using the Fiji plugin Jfilament2D [65] and the curvature of microtubules were estimated using a custom‐made python script (see Supplementary Figure S2 for analysis workflow). The obtained curve from JFilament2D was first smoothed by a parametric spline interpolation to remove noise. Then, the Menger curvature was calculated using three points spaced 120 nm apart. Curved microtubules displaying a maximum local curvature of 0.15 µm^−1^ or above were considered as bent. Bent zones of bent microtubules refer to sections of the bent microtubule with a mean local curvature above 0.2 µm^−1^. Mean curvature over the entire microtubule was estimated by averaging the local curvature of all segments of the traced microtubule. Curvature at point of breakage of breaking buckling microtubules was estimated by taking an average of the local curvature of all segments in the region spanning 500 nm around the point of breakage.

### 4.18 Estimation of the Microtubule Persistence Length

Microtubule persistence length was estimated by analyzing thermal fluctuation of microtubules. For this, microtubules were elongated from biotin‐containing microtubule seeds (attached to a streptavidin coated SiPEG‐biotin cover‐glass) using elongation buffer supplemented with 1 mM GTP and 11 µM tubulin for 15 min at 37°C. Following this the elongated microtubules were capped by flushing in a solution of elongation buffer supplemented with 0.5 mM GMPCPP and 2.5 µM tubulin and allowed to incubate at 37°C for 5 min. After washing with imaging buffer, the chamber was sealed, and microtubules were imaged every 10 s for 40 min. Persistence length was estimated by following the method reported in [25]. The filament coordinates were obtained by automatized tracing using T‐SOAX software [66]. Analysis of these coordinates (calculation of tangent angles, cosine modes and fitting) was carried out via a custom‐written python code (Refer Rig. 7f and Supplementary Figure S5i). For experiments with HEK293 lysate, 20 µg mL^−1^ of cell lysate was flushed in with the imaging buffer prior to sealing of chamber and imaging.

### 4.19 Quantification of Lateral Tubulin Incorporation and % of Tubulin Turnover

The % tubulin turnover) across all conditions was estimated from quantifications of the lateral spread (across protofilaments) as well as longitudinal spread (along the microtubule lattice length, defined as incorporation length/microtubule length) of each incorporation event. The no: of protofilaments replaced was estimated from quantifications of the amount of lateral tubulin incorporation. The amount of lateral tubulin incorporation in incorporated stretches in all datasets was estimated by using stretches of microtubule elongation (stretches of 100% green‐labeled tubulin that we occasionally observed beyond the cap‐ Refer Figure 1j, top, Supplementary Figure S1e) as a reference stretch that we assumed to possess a 13‐protofilament structure. The integrated fluorescence intensity of the incorporation, elongation stretch as well as the background was estimated using Fiji. The integrated elongation intensity (I_elongation_) and incorporation intensity (I_inc_) was obtained after subtracting the background intensity. As described in [5, 8], using the estimates of the incorporation length (FWHM), the integrated (I_total_) fluorescence intensity of the elongation stretch, length of the tubulin dimer (L = 8 nm), we estimated the fluorescence intensity of a single tubulin dimer as (I_dimer_) as I_dimer_ = I_elongation_*L /(FWHM*13). From the integral fluorescence intensity (I_inc_) of the incorporation, we estimated the number of incorporated dimers as N_inc_ = I_inc_/I_dimer_. The values of N_inc_ from five frames per incorporation stretch were averaged to obtain the amount of lateral tubulin incorporation per incorporation in each condition. The no: of protofilaments replaced was calculated as N_PF_ = (% of lateral tubulin incorporation) *13. (Finally, tubulin turnover % was computed by multiplying the mean value of the incorporation length/microtubule length (estimate of the lattice length replaced) with the average amount of lateral incorporation for each condition.

### 4.20 Categorization of Different Microtubule Bending Events in Cells

Time‐lapses of dynamic microtubules in live PtK2 cells were analyzed for quantification of microtubule bending events in cells. The bending events in cells were classified in to ‘Bent persisting’, ‘Buckling’, ‘Looping’ and ‘Breakage’ events (See Supplementary Figure S3a). A microtubule was considered to persist in the bent form without relative change in its curvature (less than 0.1–0.15 µm^−1^) (like in Figure 2b(i)) for the period of observation (5 min). Microtubules showing dynamic change in curvature (See Figure 2a,b(ii)) were classified as buckling. Buckling microtubules that were seen to adopt a loop‐like conformation were categorized as looping microtubules. We also observed relatively rare instances of microtubule breakage following bending/buckling (like in Figure 2b(iii) and Supplementary Figure S3b). A total of 4 cells from two independent experiments were analyzed.

### 4.21 Microtubule Survival

For microtubule survival experiments, time‐lapses were recorded for 40 min with a frame interval of 10 s. The % microtubule population remaining was estimated by manually counting the no: of microtubules in each frame within the same field‐of‐view every 2 min.

### 4.22 Estimation of Kinesin Motility Parameters

Gliding velocity of microtubules both in the presence and absence of HEK293 cell lysate was estimated by tracking the movement of a gliding microtubule in 10 consecutive frames using MTrackJ Fiji plugin. Kinesin motility parameters were estimated from kymographs generated from traces using the KymoResliceWide plugin.

### 4.23 Statistical Analysis

Statistical analysis was performed using GraphPad Prism software (version 9.5). To test the significance in the case of incorporation lengths and amount of lateral tubulin incorporation, Mann‐Whitney test (two‐tailed) was used as a non‐parametric alternative to a *t*‐test, as the distributions are non‐Gaussian in nature. For comparisons of incorporation frequency, incorporation length/microtubule length, unpaired‐*t*‐test was used as the distributions have similar variances and are Gaussian in nature.

## Author Contributions

La.S., R.S., S.D., and Lu.S. conceptualized and guided the project. La.S., S.N., S.D., and M.W. designed the experiments. S.N. carried out the experiments and analysis. M.G. and B.K. purified the tubulin. M.W. assisted with image and curvature analysis. C.M.A. assisted with cell lysate experiments and design of figure schematics. R.S., Lu.S., and J.B. designed, performed and analysed the simulations. La.S., S.N., and R.S. wrote the manuscript. All authors provided critical feedback.

## Funding

Lu.S., La.S., S.D., and R.S. were supported by the DFG grant SFB 1027. The work was financially supported by the European Research Council (ERC; Grant No. StG 101115795 to LaS).

## Ethics Statement

The authors have nothing to report.

## Conflicts of Interest

The authors declare no conflicts of interest.

## Supporting information




**Supporting File 1**: advs74664‐sup‐0001‐SuppMat.pdf.

**Supporting File 2**: advs74664‐sup‐0002‐MovieS1.mov.

**Supporting File 3**: advs74664‐sup‐0003‐MovieS2.mov.

**Supporting File 4**: advs74664‐sup‐0004‐MovieS3.mov.

**Supporting File 5**: advs74664‐sup‐0005‐MovieS4.mov.

**Supporting File 6**: advs74664‐sup‐0006‐MovieS5.mov.

**Supporting File 7**: advs74664‐sup‐0007‐MovieS6.mov.

**Supporting File 8**: advs74664‐sup‐0008‐MovieS7.mov.

**Supporting File 9**: advs74664‐sup‐0009‐MovieS8.mov.

**Supporting File 10**: advs74664‐sup‐0010‐MovieS9.mov.

**Supporting File 11**: advs74664‐sup‐0011‐MovieS10.mov.

**Supporting File 12**: advs74664‐sup‐0012‐MovieS11.mov.

**Supporting File 13**: advs74664‐sup‐0013‐MovieS12.mov.

**Supporting File 14**: advs74664‐sup‐0014‐MovieS13.mov.

## Data Availability

Curvature analysis code and source data underlying the main and supplementary figure plots will be made available in the Zenodo repository link: 10.5281/zenodo.16935847 upon publication. The custom simulation code is available upon reasonable request by contacting the corresponding authors.
