# Supplementary material for: Kinesin‐Induced Buckling Reveals the Limits of Microtubule Self‐Repair
Source: Adv Sci (Weinh). 2026 Mar 12;13(26):e21721. doi: 10.1002/advs.202521721 (PMC13159159; doi:10.1002/advs.202521721)
Supplement: Supplementary file 1 — Supporting File 1: advs74664‐sup‐0001‐SuppMat.pdf. [file ADVS-13-e21721-s002.pdf]

Supplementary information for: Kinesin-induced buckling reveals the limits of microtubule self-repair

Shweta Nandakumar[1], Jonas Bosche[2], Mirko Wieczorek[1], Constantin Matteo Albrecht[1], Belinda König[1], Mona Grünewald[1], Ludger Santen[2], Stefan Diez[3,4,5], Reza Shaebani[2]*, Laura Schaedel[1,6]*

Affiliations:
[1] Experimental Physics and Center for Biophysics, Saarland University, 66123 Saarbrücken, Germany
[2] Theoretical Physics and Center for Biophysics, Saarland University, 66123 Saarbrücken, Germany
[3] B CUBE - Center for Molecular Bioengineering, TUD Dresden University of Technology, 01307 Dresden, Germany
[4] Max Planck Institute of Molecular Cell Biology and Genetics, 01307 Dresden, Germany
[5] Cluster of Excellence Physics of Life, TUD Dresden University of Technology, 01062 Dresden, Germany
[6] PharmaScienceHub (PSH), 66123 Saarbrücken, Germany

*Corresponding authors (shaebani@lusi.uni-sb.de; laura.schaedel@uni-saarland.de).

This PDF file includes:
1. Supplementary text for simulation methods
2. Supplementary figures S1-S10
3. Legends for supplementary movies S1-S13
4. Supplementary references

Simulation Methods

Description of the microtubule:
Our numerical model describes the active bending of microtubules (MTs), which are represented as semi-flexible filaments subject to deformations from active forces generated by molecular motors. The energy of a given filament of length L and bending rigidity $k_{\text{bend}} = L_p k_B T$ (L_p being the persistence length of the filament) is calculated using the worm-like chain model [76, 77]:

$$E_{\text{filament}} = \frac{k_{\text{bend}}}{2} \int_0^L \left(\frac{\partial \theta(s)}{\partial s} \right)^2 ds, \tag{1}$$

where s is the contour length along the filament, $\theta(s)$ is the local tangent angle, and $\partial\theta(s)/\partial s$ is the local curvature. To simulate filament dynamics numerically, the filament is discretized into N segments of equal length ($\Delta L = \frac{L}{N} = 8\,\text{nm}$). The segments are assumed to be inextensible. The configuration of the filament is then parametrized by a set of angular coordinates θ_i corresponding to the orientation of each segment with respect to the x-axis. The discretized version of the Hamiltonian becomes:

$$E_{\text{filament}} = \frac{k_{\text{bend}}}{\Delta L} \sum_{i=1}^{N} \Big(1 - \cos\left(\theta_i - \theta_{i-1}\right) \Big). \tag{2}$$

The angle of the first segment is fixed at $\theta_0 = 0$. For a straight filament aligned with the x-axis, $E_{\text{filament}} = 0$. The position of each node $\mathbf{x}_i$ is given by:

$$\mathbf{x}_i = \sum_{j=0}^{i} \Delta L \begin{pmatrix} \cos(\theta_j) \\ \sin(\theta_j) \end{pmatrix}. \tag{3}$$

Active cross-linkers:

We model the action of active molecular motors stochastically. Motors (modeled as cross-linkers) attach from a 2D grid underlying the filament. Each cross-linker on this grid can stochastically attach to a node on the filament. Once attached, the cross-linker applies a force like a linear spring:

$$E_{\text{crosslink}} = \frac{k_{\text{spring}}}{2} \sum_{i=1}^{n} ||\mathbf{x}_{\text{link},i} - \mathbf{x}_{\text{grid},i}||^2, \tag{4}$$

where k_{spring} is the spring constant, $\mathbf{x}_{\text{link},i}$ is the node between adjacent segments of the filament, and $\mathbf{x}_{\text{grid},i}$ is the fixed point of the cross-linker on the grid. If the node is within a attachment radius d_{attach}, unbound motors can attach to the filament with rate ω_{a}. While the attachment rate can be force-dependent in general (i.e. $\omega_{\text{a}}(F) = \omega_{\text{a,0}} \exp\left(-\frac{|F|}{f_{\text{a}}}\right)$ with f_{a} being the characteristic attachment force), here we report the results for a constant ω_{a}.

In contrast, the detachment rate depends on the force:

$$\omega_{\text{d}}(F) = \omega_{\text{d,0}} \exp\left(\frac{|F|}{f_{\text{d}}}\right), \tag{5}$$

where f_{d} is the characteristic detachment force and $|F|$ is the absolute value of the force exerted on the filament by the motor. To avoid unrealistically large forces and numerical instabilities, a maximum motor extension length of $40\,\text{nm}$ was imposed. Once attached, motors can walk along the filament from the fixed end toward the free end. The step size d_{h} is constant ($d_{\text{h}} = \Delta L = 8\,\text{nm}$). Similar to the detachment rate, the hopping rate is also force-dependent. If the force and motion direction are aligned the hopping rate is given by

$$\omega_{\text{h}}(F) = \min\left[2\,\omega_{\text{h,0}}, \omega_{\text{h,0}}\left(1 + \frac{|F|}{f_{\text{s}}}\right)\right], \tag{6}$$

otherwise

$$\omega_{\text{h}}(F) = \max\left[0, \omega_{\text{h,0}}\left(1 - \frac{|F|}{f_{\text{s}}}\right)\right], \tag{7}$$

where f_s is the stall force and $\omega_{h,0}$ is the load-free hopping rate. In the simulation, we assume a separation of timescales: the relaxation of the filament is much faster than the timescale of motor dynamics. Therefore, after each motor update (attachment, detachment, or stepping), the shape of the filament is re-equilibrated. Nevertheless, this equilibrated configuration will change in the next time step since the status of motors evolves with time (including new attachments, new detachments, and motor position updates). The choice of the initial nodes to attach motors to the filament and their updates because of motor dynamics are carried out using the Gillespie algorithm. After each step, the energy of the system is minimized using a gradient descent method.

The mean force per motor is computed by evaluating the elastic stretching force of each motor, modeled as a linear spring. Specifically, for each active motor, we calculate the product of the spring constant and the displacement vector between the position of the motor on the filament and its fixed attachment point on the grid. The mean force per motor is then obtained by averaging these forces over all active motors bound to the filament. To determine the mean force per discrete filament segment, we first sum the force vectors from all motors that are attached to the same node of the filament. This vector sum represents the total force exerted on that segment. Finally, we compute the average over all such segments along the filament to obtain the mean force per discrete segment. To quantify the local curvature of the filament, we assign a curvature value to each internal node based on the positions of three successive nodes along the discretized filament. For each triplet of adjacent nodes, we compute the radius of the circle that passes through all three points. The local curvature at the central node is then defined as the inverse of this radius.

To investigate the spatial relationship between local curvature and force along the filament, we computed the cross-correlation function $\text{Corr}(\Delta x)$ between the local curvature $c(x,t)$ and the local force $f(x \pm \Delta x, t)$, evaluated at spatially offset positions along the filament. This function quantifies the correlation between curvature at position x and force at a neighboring position $x \pm \Delta x$, yielding a value between 1 and -1, indicating positive and negative correlation, respectively.
The cross-correlation was calculated using the standard Pearson correlation coefficient formula: $\text{Corr}(\Delta x) = \frac{\langle (c(x,t) - \langle c \rangle)(f(x \pm \Delta x, t) - \langle f \rangle) \rangle}{\sigma_c \sigma_f}$. Here, the parameters enclosed within $\langle \rangle$ are obtained by averaging over filament segments at a given time t, and σ_c and σ_f are the standard deviations of the curvature and force, respectively.

Estimation of the steady-state number of bound motors:

To compute the steady-state number of motor proteins attached to a microtubule filament, we model motor binding and unbinding as a stochastic two-state Markov process influenced by an external force. Motors bind with a force-dependent attachment rate $\omega_a(f) = \omega_{a,0} \exp\left(-\frac{|F|}{f_a}\right)$ and detach with a rate $\omega_d(f) = \omega_{d,0} \exp\left(\frac{|F|}{f_d}\right)$ (see Eq. 5). $|F|$ is the absolute value of the average force exerted on the filament by the motors at different motor densities (refer Fig 6i). By analyzing the Markov chain balance of stochastic binding and unbinding events, the steady-state fraction of available motors which remain bound can be obtained as $\frac{1}{1 + \frac{w_{d,0}}{w_{a,0}} \exp(|F|(1/f_d + 1/f_a))}$. Given a surface motor density ρ, the number of motors geometrically available to interact with the filament is approximately $\sqrt{\rho}$, per micrometer.
Therefore, the steady-state number of motors that remain bound yields

$$N_{\text{steady}} = \frac{\sqrt{\rho}}{1 + \frac{w_{d,0}}{w_{a,0}} \exp(|F|(1/f_d + 1/f_a))}$$

In case of a constant (force-independent) ω_a, as in our simulations, the relation for the steady-state number of bound motors reduces to

$$N_{\text{steady}} = \frac{\sqrt{\rho}}{1 + \frac{w_{d,0}}{w_{a,0}} \exp(|F|/f_d)}$$

Estimation of forces acting on statically bent microtubules in experiments:

To estimate the force exerted on statically bent microtubules observed in experiments (Refer Fig 1c, right), we model the microtubule as a semiflexible filament confined to two dimensions and clamped at one end [41]. The full shape of the microtubule is extracted from microscopy images. An external force with unknown x- and y-components is assumed to act at the free end. Our goal is to determine the force that best reproduces the observed static configuration under these boundary conditions. For the numerical simulations, the filament is assumed to have a given bending rigidity and is discretized into segments, as described above. To estimate the applied force, we fix the position and orientation (tangent angle) at the fixed end, the components of a trial force at the free end and obtain the equilibrium configuration of the filament. If the applied trial force differs from the true value, the resulting shape evolves away from the experimentally observed configuration and the position of the free end changes. Thus, for each trial force we compute the Euclidean distance between the simulated and observed position of the free end. By scanning across a range of trial force values, we identify the one that minimizes this distance as the optimal force reproducing the observed filament shape under the given constraints.

Supplementary table S1:

Parameter	Symbol	Value	Unit	Reference
Thermal persistence length	L_p	[1,10]	mm	This study (Suppl. Fig. S5i)
Default thermal persistence length	$L_{p,0}$	5	mm	This study (Suppl. Fig. S5i)
Microtubule length	L	[5,15]	μm	This study
Default microtubule length	L_0	10	μm	This study (Suppl. Fig. S5h)
Microtubule discrete units	ΔL	8	nm	[41],[42]
Hopping step size	d_h	8	nm	[41],[42]
Attachment rate	ω_a	[1,10]	s^{-1}	[42],[67],[68]
Default attachment rate	$\omega_{a,0}$	5	s^{-1}	[42],[67],[68]
Detachment rate	$\omega_{d,0}$	0.5	s^{-1}	This study (Suppl. Fig. S5g), [67], [68]
Hopping rate	$\omega_{h,0}$	81	s^{-1}	[67]
Motor density	ρ	[100,500]	μm^{-2}	This study
Default motor density	ρ_0	400	μm^{-2}	This study (Suppl. Fig. S5b)
Spring constant	k	0.0003	N/m	[69]
Detachment force	f_d	3	pN	[70],[71]
Attachment force	f_a	3	pN	[72],[73]
Motor stall force	f_s	6	pN	[74],[75]

Supplementary table S1: Parameters used in numerical simulations of microtubule deformation under active forces. For experimental data values obtained from experiments, please refer **Suppl. Fig. S5.**

Supplementary figure 1

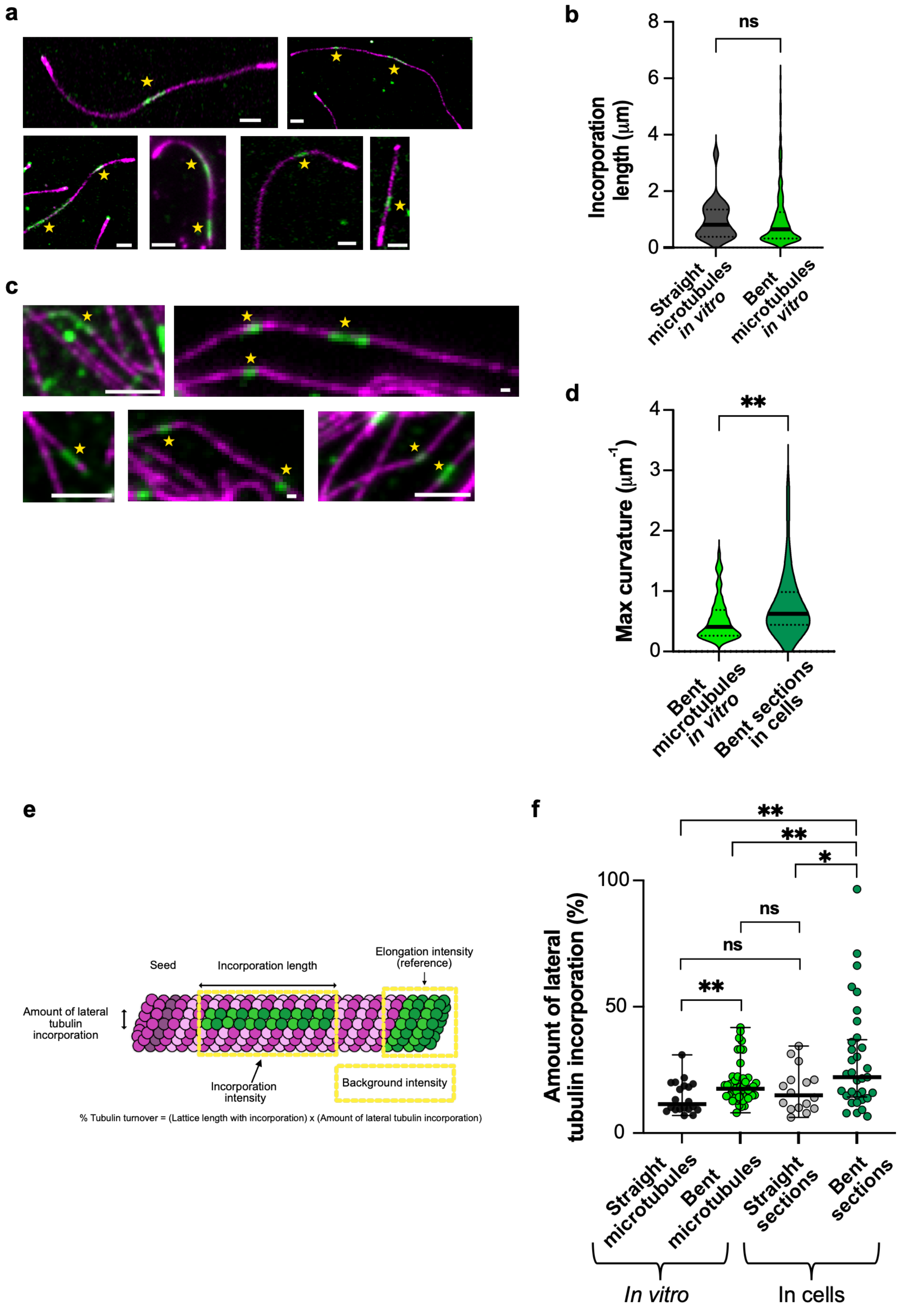

Supplementary Figure S1: a, Additional examples of incorporations (marked with a yellow star) in static straight and bent microtubules *in vitro* (scale bar: 2 µm) **b**, Comparison of incorporation length in static straight vs static bent microtubules *in vitro* (global). Black line

represents the median and dotted lines, the interquartile range. $p = 0.2905$ (ns, not significant) using Mann-Whitney test. Total length of microtubule analyzed in both cases: 1,104 µm ($n = 23$ incorporations for straight microtubules and $n = 263$ incorporations for bent microtubules) **c**, Additional examples of incorporations (marked with a yellow star) in straight and bent sections in cells (scale bar: 2 µm) **d**, Comparison of curvature of static bent microtubules *in vitro* vs bent sections in cells. Black line represents the median and dotted lines, the interquartile range. $p = 0.027$ using an unpaired t-test ($n = 127$ *in vitro*; $n = 36$ in cells). **e,** Schematic showing the different parameters estimated to quantify the number of protofilaments and % tubulin turnover in a microtubule with incorporation (See **Methods). f,** Higher amount of lateral tubulin incorporation in bent microtubules, both in cells and *in vitro*. Three independent experiments were analyzed for each condition. $n = 21$ incorporations for straight microtubules (*in vitro)*, $n = 51$ incorporations for bent microtubules (*in vitro)*, $n = 16$ incorporations for straight sections (cells) and $n = 32$ incorporations for bent sections (cells). Black lines represent the median and error bars represent the interquartile range. Statistical test used: Mann-Whitney test. $p = 0.0391$ (straight sections, cells -bent sections, cells); $p = 0.0024$ (straight microtubules, *in vitro*-bent microtubules, *in vitro*); $p = 0.0095$ (bent sections, cells-bent microtubules, *in vitro*); $p = 0.2411$ (not significant; straight sections, cells-straight microtubules*, in vitro*); $p = 0.2163$ (not significant; straight sections, cells-bent microtubules, *in vitro*) and $p = 0.2411$ (not significant; straight sections, cells-straight microtubules, *in vitro*).

Supplementary figure 2

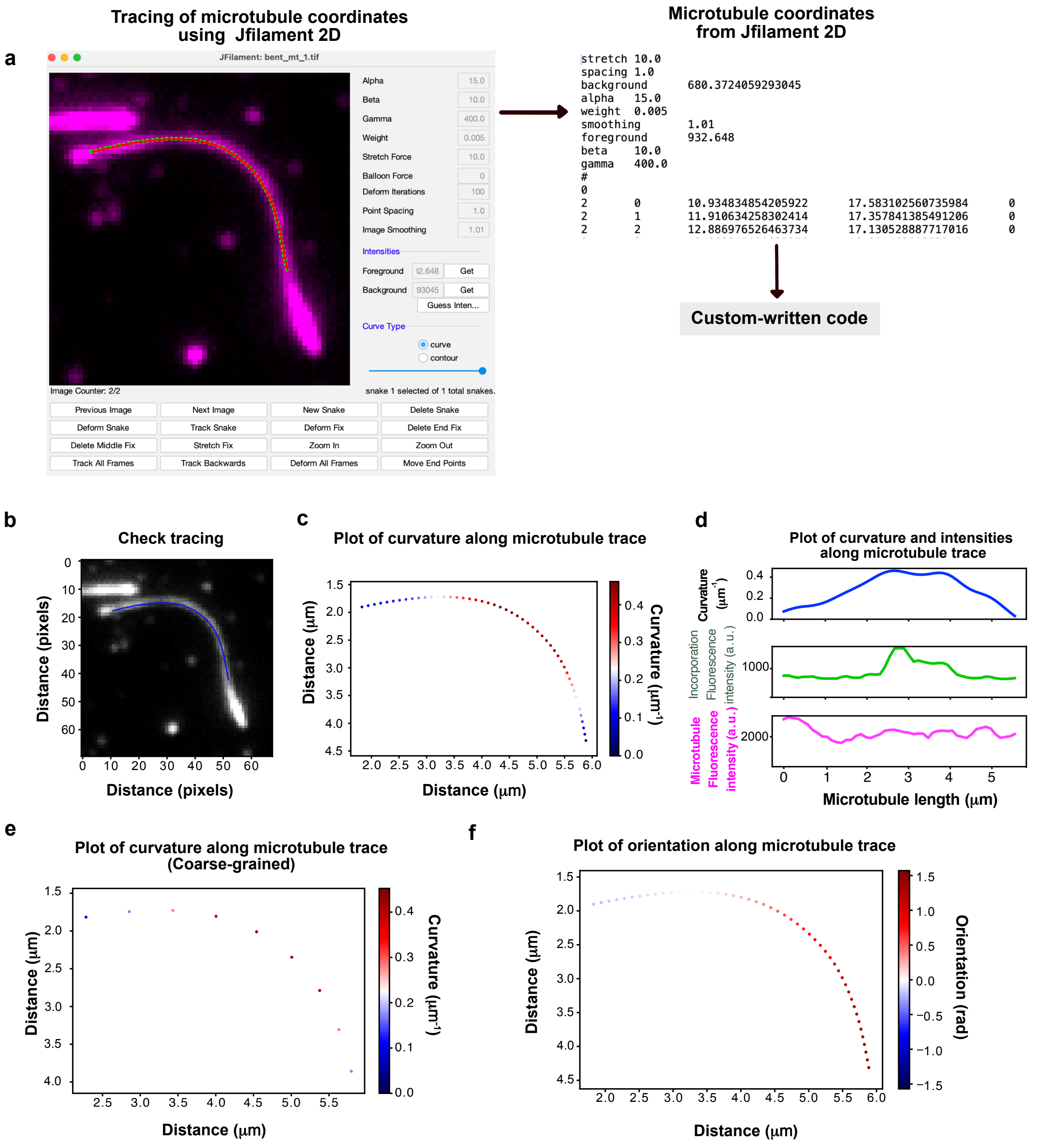

Supplementary Figure S2: Curvature analysis workflow. a, Microtubules were traced using the Fiji plugin JFilament 2D. The obtained coordinates were used to calculate the curvature and to plot the fluorescence intensities along the microtubule arclength using a custom-written code. **b,** *Output 1 from code:* Image of traced microtubule superimposed on the original microtubule image to check for accuracy of tracing. Checking the accuracy of tracing might be necessary if the obtained curve from JFilament 2D was additionally smoothed in the code. **c,** *Output 2 from code:* Plot of curvature (color-coded) along the traced microtubule **d,** *Output 3 from code:* Plot of curvature (μm^{-1}) and fluorescence intensities (a.u.) of both microtubule and incorporation channel along the microtubule length (μm). **e,** *Optional output 4 from code:* Plot of coarse-grained curvature (color-coded) along the microtubule segments. **f,** *Optional output 5 from code:* Plot of orientation (color-coded) along the traced microtubule. Microtubules showing a local curvature higher than 0.15 μm^{-1} were considered as bent.

Supplementary figure 3

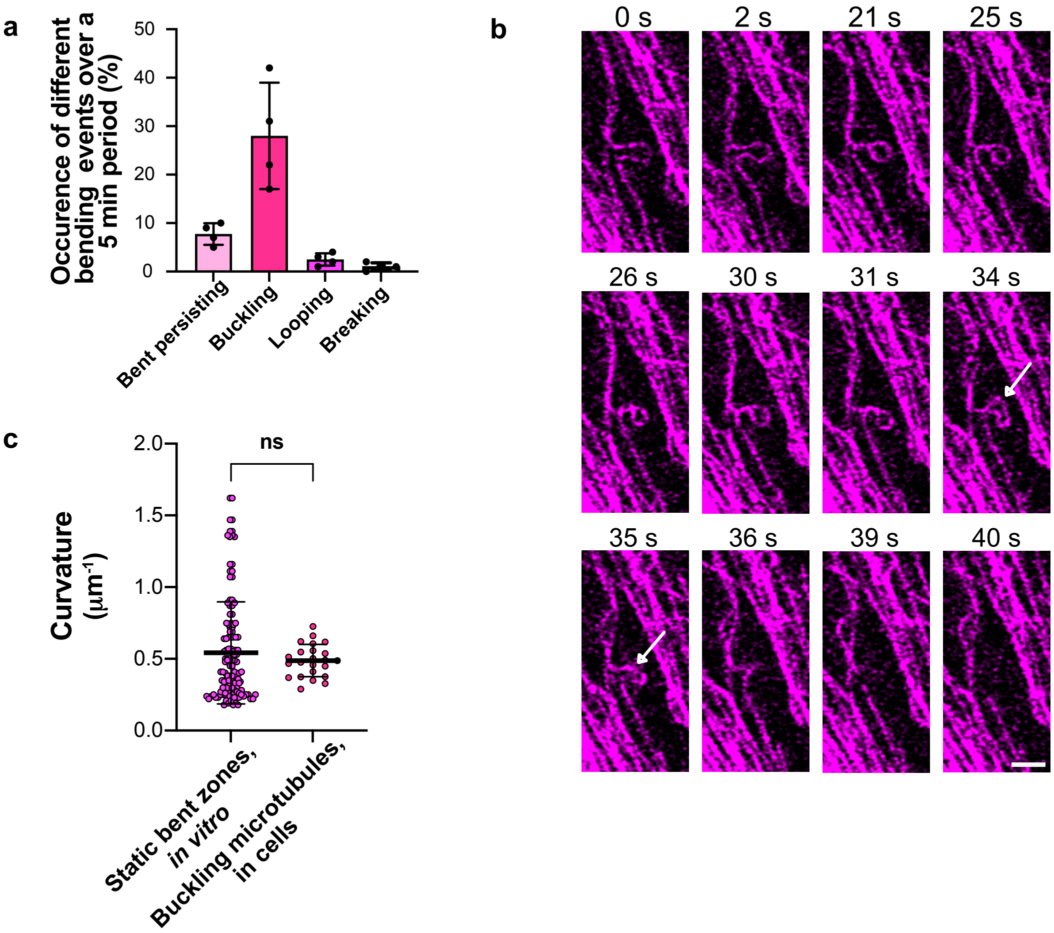

Supplementary Figure S3: a, *Quantification of different microtubule bending events* over a 5 min period in live PtK2 cells (with an endogenous tubulin-eGFP tag; represented in magenta). Error bars represent the S.D. Black dots represent individual cells. n = 4 cells analyzed from two independent experiments. **b,** Time-lapse sequence showing breakage at high curvatures (marked by white arrow) in a looping microtubule in a live PtK2 cell. Scale bar: 2 µm. **c,** Comparison of curvature between the bent zones of static bent microtubules (*in vitro* analyzed in **Fig 1d**) and buckling microtubules (in cells, analyzed in **Fig 2g**). Black line represents the mean and error bars represent the S.D. (n = 127 microtubules from static bent zones *in vitro* and n = 23 buckling microtubules, *in vitro* from three independent experiments). p = 0.4721 (not-significant; ns) using unpaired-t-test.

Supplementary figure 4

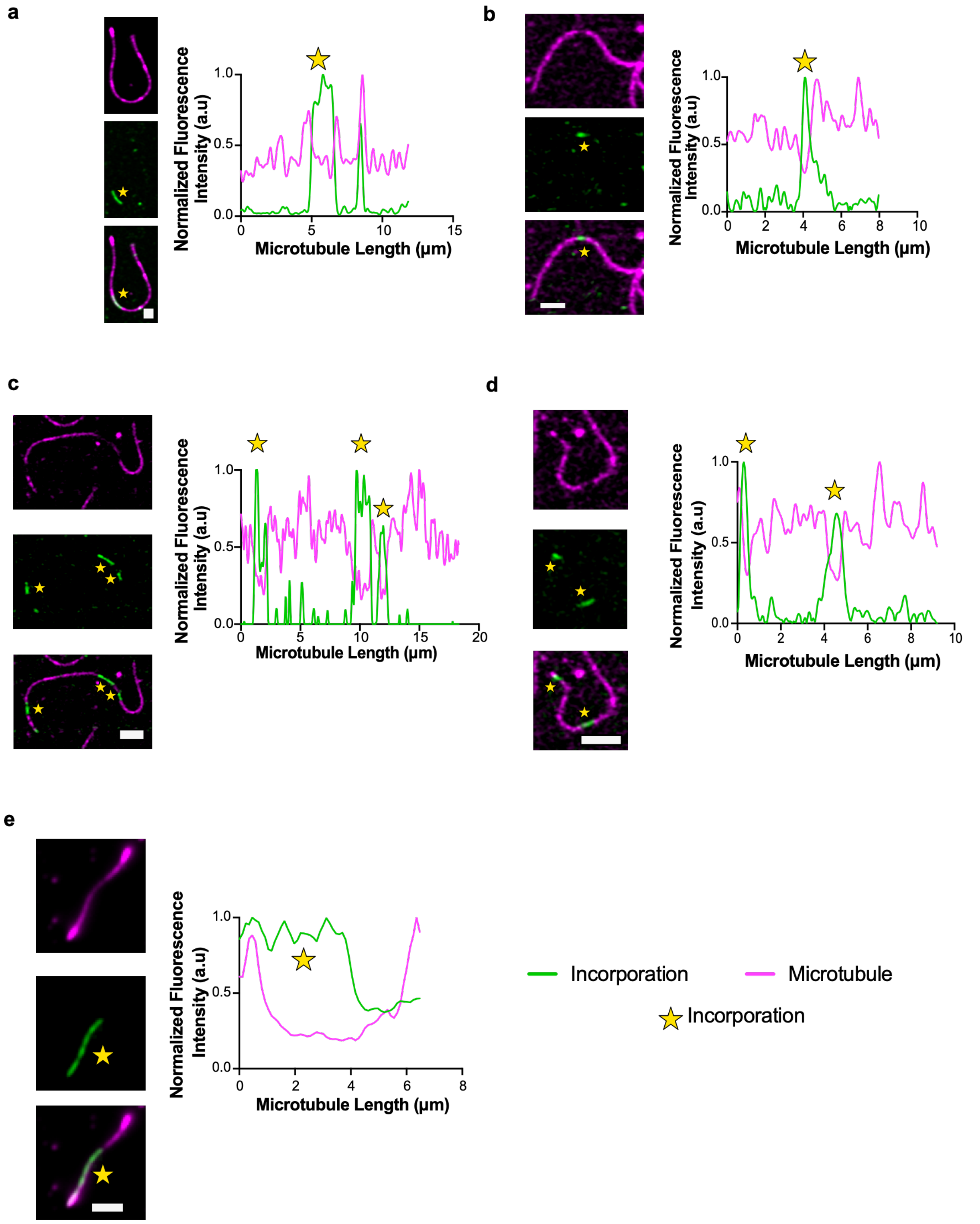

Supplementary Figure S4: a-e, Exemplary images with fluorescence intensity line scans of incorporations in buckling microtubules. We frequently observe a dip in the fluorescence intensity of the microtubule lattice (in magenta) at incorporation sites, indicating that a significant proportion of the lattice has been replaced. Scale bars: 2 µm.

Supplementary figure 5

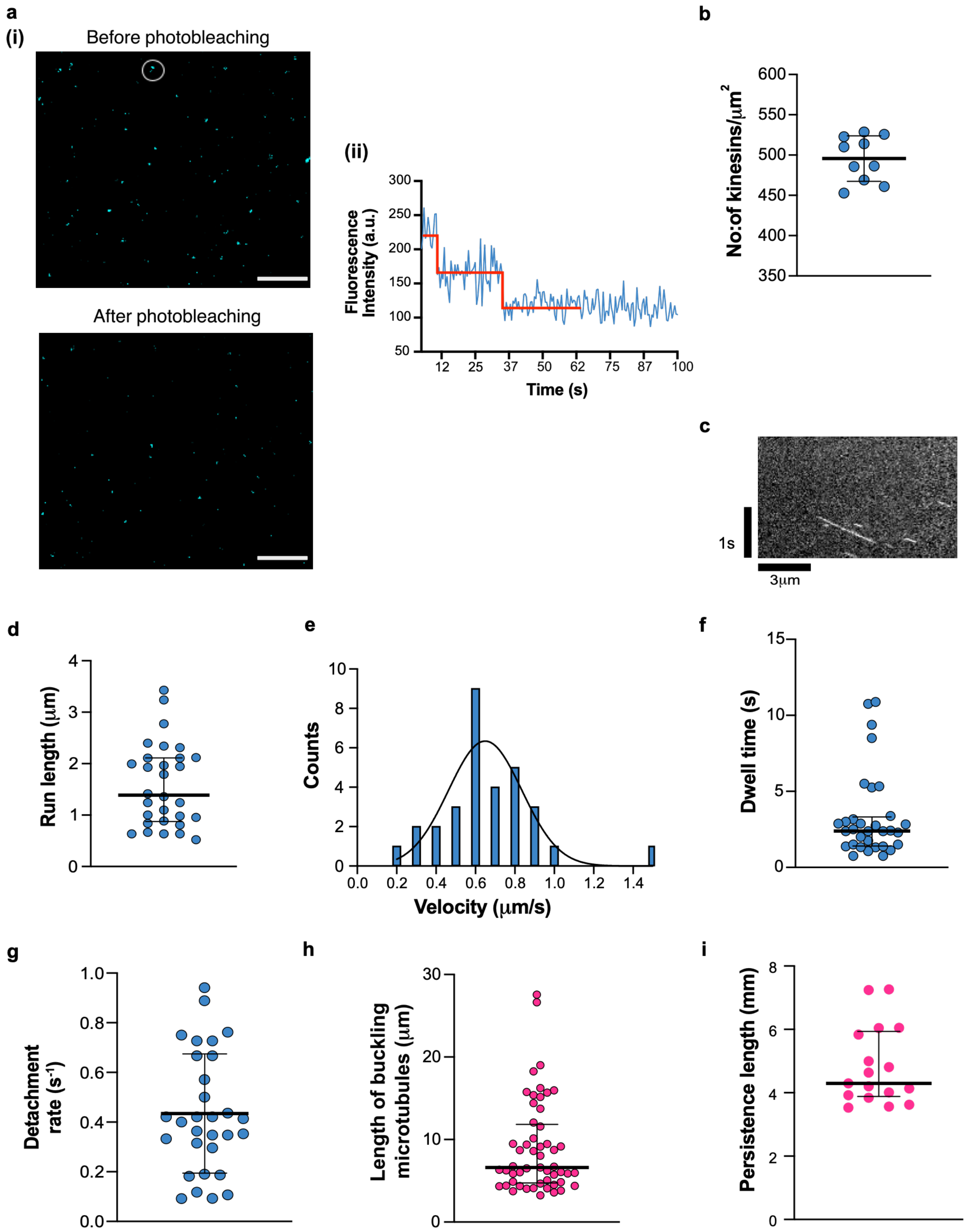

Supplementary Figure S5: *Data underlying experimental parameters for simulations: Single molecule photo-bleaching to estimate kinesin surface density:* **a,(i)** Example image of kinesin-1-GFP (represented in cerulean blue) molecules at 350 pM before and after photobleaching using a laser power of 130 mW in streaming mode, with 500 ms exposure time. White circle indicates a kinesin-1-GFP molecule that disappears over time due to photobleaching. Scale bar: 5 μm. **a,(ii)** Trace showing two-step photobleaching of the molecule circled in a(i). Red line is overlaid for better visualization of the

two-step drop in fluorescence intensity **b,** Plot showing distribution of the surface density of kinesin-1 estimated from photobleaching experiments. Black line represents the mean and bars represent the S.D (n = 10 traces from 10 spots were analyzed from two independent experiments). *Single molecule motility studies to determine kinesin motility parameters:* **c,** Exemplary kymograph showing motile kinesin-1 molecules. **d,** Distribution of run length of kinesin-1 molecules. Black lines represent the median and the bars represent the interquartile range. **e**, Distribution of the velocities of kinesin-1 molecules. **f**, Distribution of dwell time of kinesin-1 molecules on microtubules. Black line represents the median and bars represent the interquartile range. **g**, Distribution of detachment rate of kinesin-1 molecules. Black lines represent the mean and bars represent the S.D. For the data underlying **Suppl. Fig. S5d-g**, n = 32 kymographs from 13 microtubules from two independent experiments were analyzed. **h,** Distribution showing length of buckling microtubules. Black lines represent the median and bars represent the interquartile range. (n = 53 microtubules from six independent experiments). **i,** Persistence length of microtubules from thermal fluctuation experiments (see **Methods**). Black lines represent the median and bars represent the interquartile range (n = 17 microtubules from three independent experiments).

Supplementary figure 6

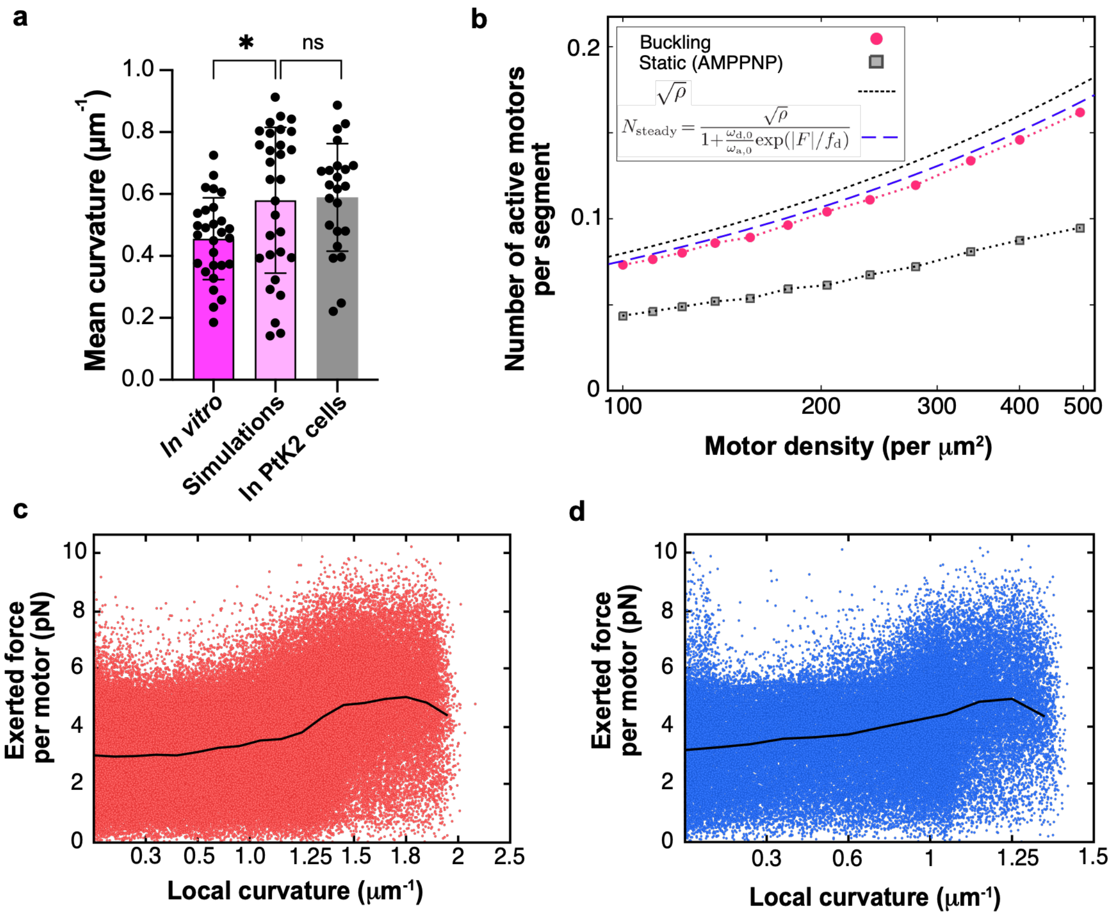

Supplementary Figure S6: a, *Comparison of mean curvature of buckling microtubules in experiments vs simulations vs in PtK2 cells. In vitro* (n = 28 timepoints, *in vitro* from 4 buckling microtubules from three independent experiments) and n = 23 timepoints from 4 PtK2 cells (analyzed from 2 independent experiments). Simulation results using a motor density of ρ = 494 µm^{-2}, microtubule persistence length, L_p = 6 mm, and microtubule length, L = 10 µm. Error bars represent the S.D. p = 0.0174 (*in vitro*-simulations) and p = 0.8786 (simulations- in PtK2 cells) using unpaired t-test. **b**, *Lin-log plot of number of active motors per segment in a simulated buckling microtubule as a function of motor density.* The steady-state number of motors bound per microtubule segment can be estimated by analyzing the Markov chain balance of stochastic binding and unbinding events, yielding a value for N_{steady} (see SI simulation methods for details). In this equation, |F| is the absolute value of the average force exerted on the filament by the motors (for both buckling and AMPPNP conditions) at different motor densities (See **Fig 6i** and **Suppl. Fig. S7c**). In practice, the actual number of motors on the filament is slightly lower than this theoretical estimate, as the system remains in a transient dynamical regime and motor traffic along the filament has not yet reached equilibrium. *Scatter plots of the force exerted by each motor vs local curvature*, shown for a: **c**, soft microtubule with L_p = 1 mm (red dots) and **d**, stiff microtubule with L_p = 10 mm (blue dots). Other parameters include: L = 5 µm, ρ = 400 µm^{-2}. Black line represents average values computed by binning the local curvature. Each plot includes data from 10^5 microtubule samples.

Note: Although the motor force values in the above scatter plots (**Suppl. Fig. S6c and S6d**) span a broad range (even beyond 10 pN), most data points cluster at lower forces. The observed high forces occur due to the large ensemble size in simulations (10^5 microtubule samples with ~10^2 motors along each). This enables us to observe statistically rare outcomes that would be difficult to capture in typical experimental datasets.

Supplementary figure 7

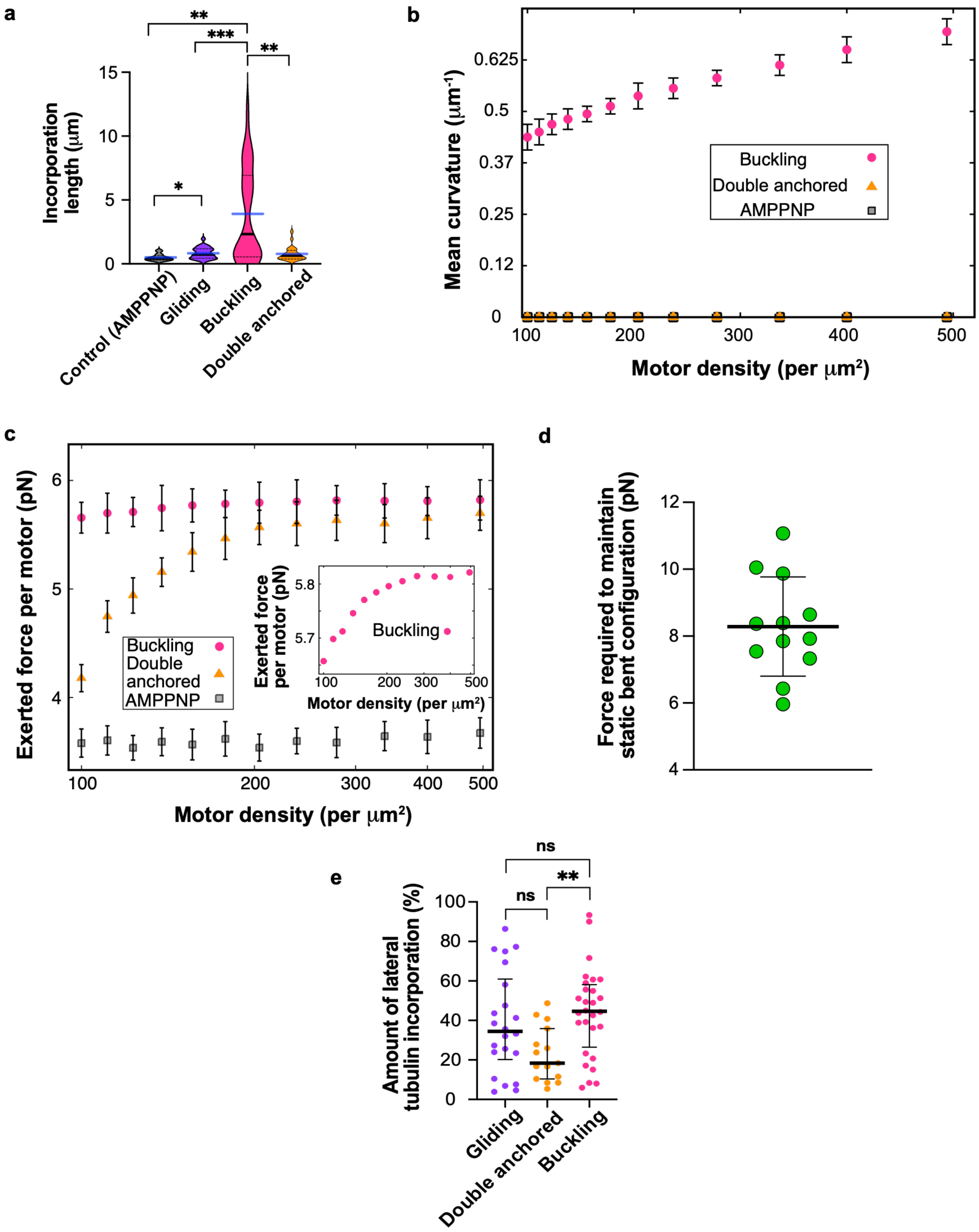

Supplementary Figure S7: a, *Incorporation length across different conditions*- control (AMPPNP), gliding, buckling and double-anchored microtubules. Black lines represent median, dotted lines represent the interquartile range and blue line represents the mean from three independent experiments in each case, comprising 98, 139 and 110 μm of the total microtubule length analyzed for double-anchored microtubules. For microtubule lengths analyzed in all other conditions, refer **Fig 3g** captions. p = 0.3442 (double anchored-gliding); p = 0.0012 (double-anchored -buckling); p = 0.0018 (buckling-control), p = 0.0172 (gliding-control), and p = 0.0005 (gliding-buckling) using Mann-Whitney test. Comparison between all other conditions are non-significant. **b,** *Effect of motor arrangement and*

mobility on microtubule dynamics. Simulations compare three configurations: (pink circles) motors distributed across the surface leading to buckling, (orange triangles) motors arranged linearly beneath a straight microtubule (double-anchored), and (gray squares) in the presence of AMPPNP. Mean curvature of each discretized node of the microtubule is plotted as a function of motor density for all three cases. Microtubule parameters: L = 10 μm and L_p = 5 mm. Data are averaged over time and across five microtubules. **c,** *Mean force exerted per motor on a microtubule as a function of motor density in simulations* for buckling, double anchored and AMPPNP conditions. Microtubule parameters: L = 10 μm and L_p = 5 mm. The inset shows the same data as the buckling case in the main figure panel in the same figure, but over a narrower force range to better highlight the increasing trend. **d,** *Force (along the entire microtubule) required to maintain static bent microtubules in the bent configuration*. Refer to SI simulation methods for details. Black lines represent mean and S.D. (n = 12 static bent microtubules were analyzed from experimental data from three independent experiments). **e,** Higher amount of lateral tubulin incorporation in buckling microtubules. Black lines represent the median and bars represent the interquartile range. Statistical test used: Mann-Whitney test. p = 0.42, not significant (gliding-buckling); p = 0.1049, not significant (gliding- double-anchored); p = 0.0019 (double-anchored -buckling). 98, 139 and 110 μm of the total microtubule length analyzed for double- anchored microtubules. For microtubule lengths analyzed in all other conditions, refer **Fig 3g** captions. Data cumulative for three independent experiments in each condition.

Supplementary figure 8

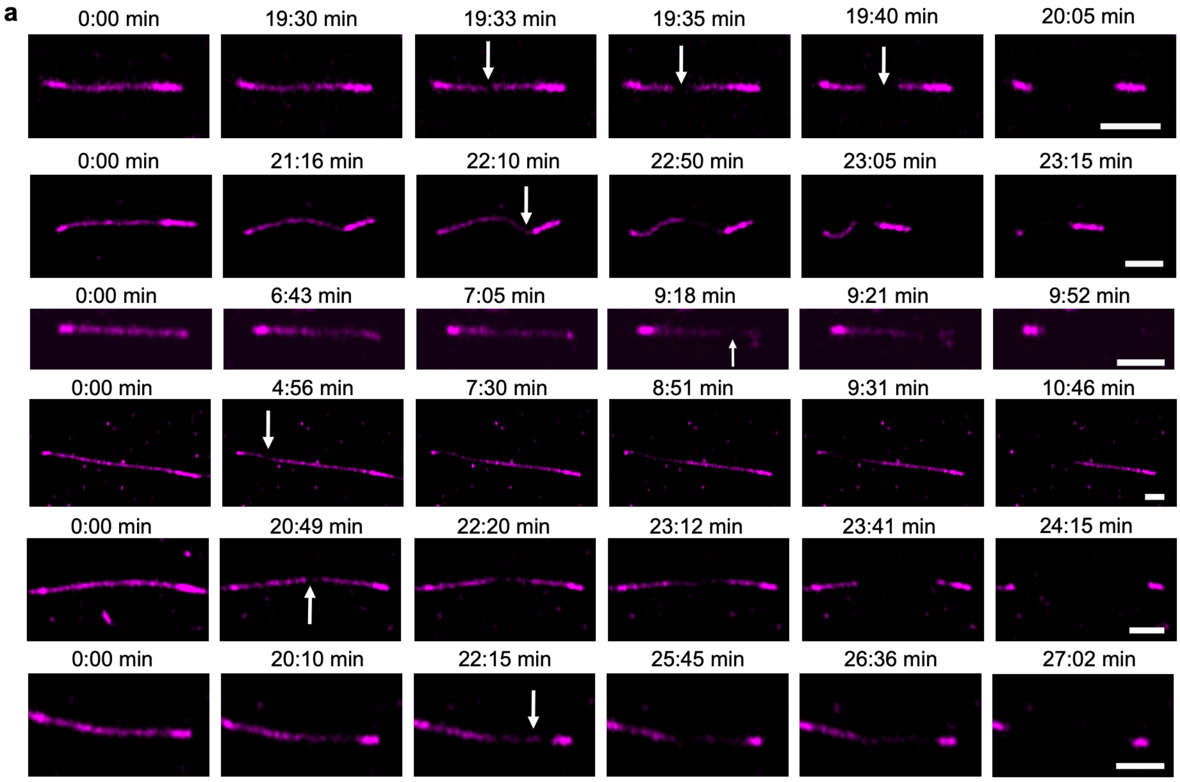

Supplementary Figure S8: a, Example images showing location and time of breakage in double-anchored microtubules. Scale bar: 2 μm.

Supplementary figure 9

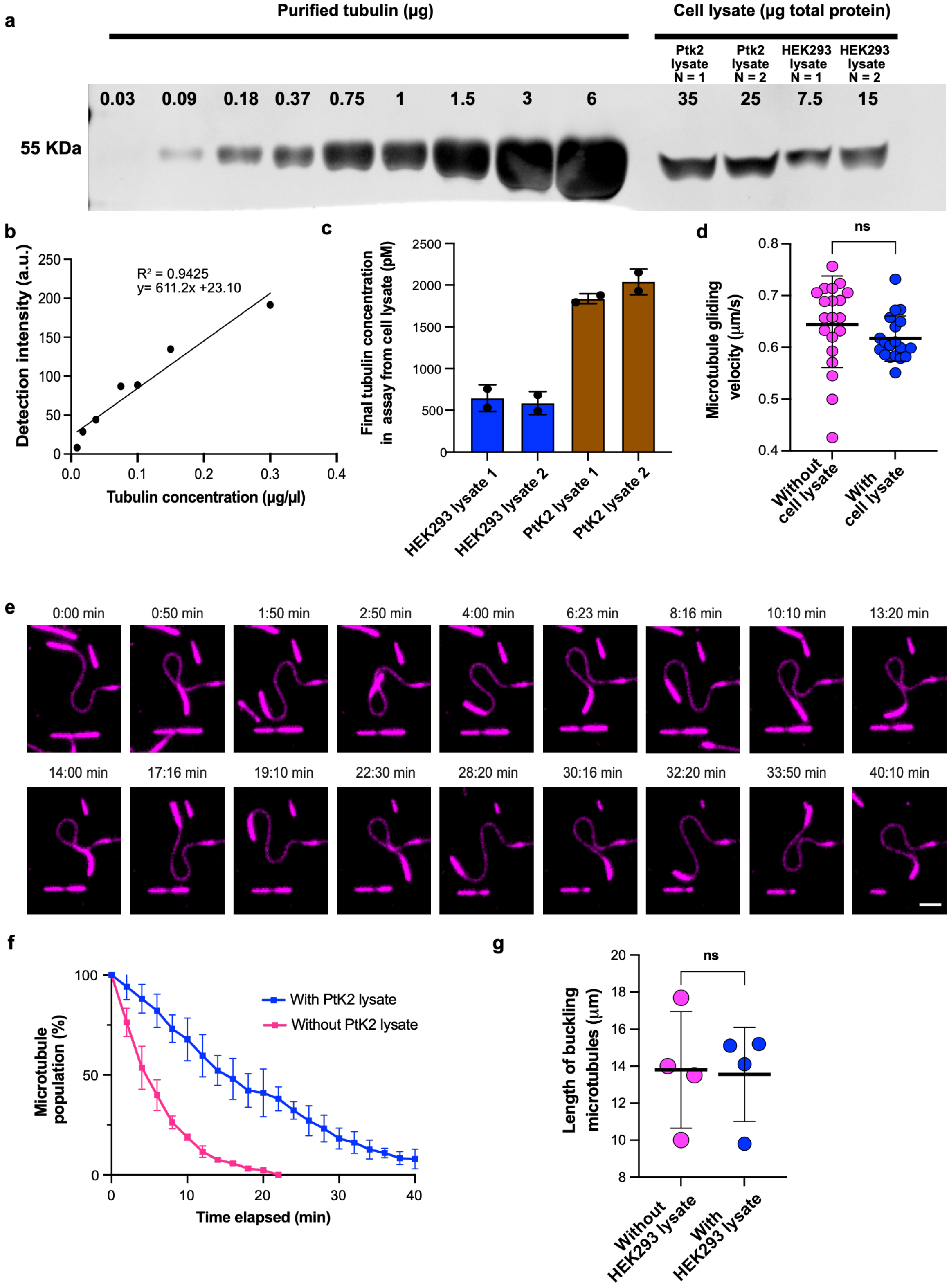

Supplementary Figure S9: a, Concentration of tubulin in cell lysates was estimated using quantitative western-blot analysis. Known concentrations of purified tubulin (0.03-6 μg) were loaded as standards with different dilutions of 2 independent lysates of HEK293 and PtK2 cells. **b**, Standard curve: Plot of intensities of purified tubulin bands in the immunoblot in **9a** vs the corresponding tubulin concentration in μg μL^{-1}. **c,** The standard curve in **b** was used to determine the tubulin content in the buckling assays

shown in **Fig 7a-c** (HEK293 cells) and **9e,f** (PtK2 cells). Concentration values were obtained from 2 immunoblots from 2 lysates of HEK293 and PtK2 cell lysates. **d,** *Comparison of microtubule gliding velocity* with and without the addition of 20 μg mL^{-1} HEK293 cell lysate. Black lines represent mean and error bars represent the S.D. $p = 0.2146$ (not significant; ns) using unpaired t-test ($n = 19$ microtubules analyzed in each condition, from two independent experiments). **e,** Timelapse sequence showing buckling microtubule in the presence of 60 μg mL^{-1} PtK2 cell lysate. Scale bar: 2 μm. **f,** Buckling microtubules survive longer in presence of PtK2 cell lysate. Comparison of the percentage of the microtubule population remaining (in the absence of free tubulin) over time in the case of buckling microtubules both with and without 60 μg mL^{-1} PtK2 cell lysate ($n > 100$ microtubules analyzed in each condition from two independent experiments from 2 independent lysates). The symbols and error bars indicate mean ± S.D respectively. **g,** *Length of buckling microtubules* compared for estimating mean curvatures of buckling microtubules with and without the addition of 20 μg mL^{-1} HEK293 cell lysate (Refer **Fig 7d**). $p = 0.9054$ (not significant; ns) using unpaired t-test ($n = 26$ frames analyzed from 4 microtubules in each condition from three independent experiments).

Supplementary figure 10

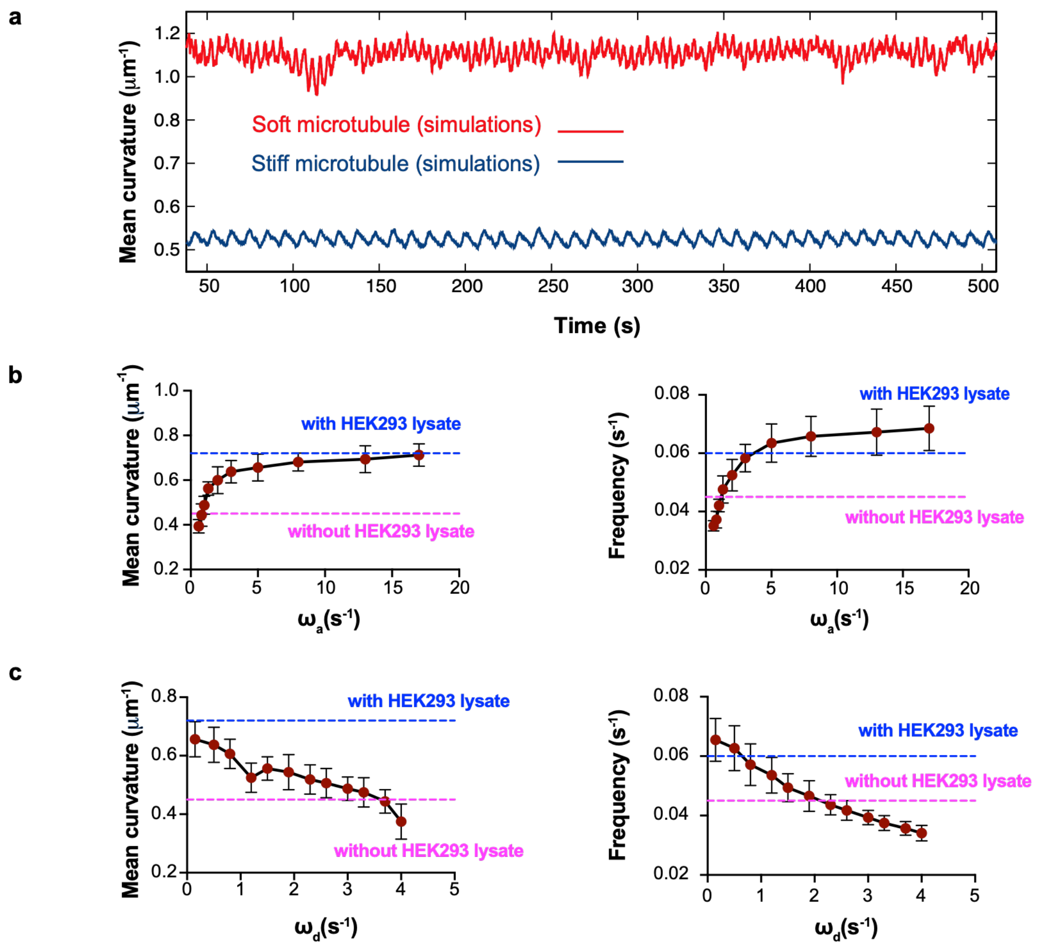

Supplementary Figure S10: a, Effect of microtubule stiffness on mean curvature. Examples of mean curvature evolution over time in simulations. The behavior of a soft microtubule (in red) with L_p = 1 mm is compared to a stiff microtubule (in dark blue) with L_p = 10 mm (slight bending phase). Other parameter values are L = 10 μm and ρ = 400 μm^{-2}. Mean curvature (left) and oscillation frequency (right) as a function of the motor attachment rate (ω_a), in **10b** and motor detachment rate (ω_d), in **10c** for a microtubule of L = 10 μm and L_p = 5 mm. The symbols and error bars indicate mean ± S.D respectively. Blue and pink dotted lines represent experimentally determined values with and without HEK293 lysate respectively.

Legends for supplementary movies:

Supplementary movie S1: Different microtubule bending events in live PtK2 cells. Videos showing dynamic buckling, microtubules persisting in the bent shape, loop formation and breakage in PtK2 cells (endogenous tubulin-eGFP tag; represented here in magenta). Movies are at 10 fps.

Supplementary movie S2: Different microtubule bending events recaptured in our dynamic buckling assay. Using our in vitro assay, we recapture microtubule behaviors as seen in cells- regular flagella-like oscillations, loop formation, beating as well as pivoting. Movies are at 20 fps.

Supplementary movie S3: Trace of mean curvature over time (left) of a microtubule (right) as it buckles. Movie is at 20 fps.

Supplementary movie S4: Gliding microtubule with incorporation. Movie is at 5 fps.

Supplementary movie S5: Buckling microtubule with incorporation. Movie is at 5 fps.

Supplementary movie S6: Looping microtubule with incorporation. Movie is at 20 fps.

Supplementary movie S7: Buckling microtubules in vitro breaking in the presence of 5 μM unlabeled free tubulin. Movies are at 20 fps.

Supplementary movie S8: Compilation of buckling and looping microtubules breaking at points of high curvature. Movies are at 20 fps.

Supplementary movie S9: Evolution of microtubule shape in the buckling regime during simulations. The motor density is set to ρ = 278 per μm^2, with all other parameters at their default values as listed in Suppl. Table 1.

Supplementary movie S10: Evolution of microtubule shape in the loop and knot formation regime during simulations. The motor density is set to ρ = 494 per μm^2, with all other parameters at their default values as listed in Suppl.Table 1.

Supplementary movie S11: Evolution of microtubule shape in the beating regime during simulation. The motor density is set to ρ = 123 per μm^2, with all other parameters at their default values as listed in Suppl.Table 1.

Supplementary movie S12: Timelapse of double-anchored microtubule subjected to pulling action of motors in a double-anchored assay. Movie is at 20 fps.

Supplementary movie S13: Comparison of buckling microtubule- in the absence of (left).vs in the presence of 20 μg mL^{-1} HEK293 wild type lysate (right). Movies are at 20 fps.

Supplementary references:

[67] M. Rank and E. Frey, “Crowding and Pausing Strongly Affect Dynamics of Kinesin-1 Motors along Microtubules,” Biophysical journal, vol. 115, no. 6, pp. 1068-1081, 2018.

[68] M. J. I. Müller, S. Klumpp and R. Lipowsky, “Tug-of-war as a cooperative mechanism for bidirectional cargo transport by molecular motors,” Proceedings of the National Academy of Sciences of the United States of America, vol. 105, no. 12, pp. 4609-4614, 2008.

[69] C. M. Coppin , D. W. Pierce, L. Hsu and R. D. Vale, “The load dependence of kinesin’s mechanical cycle,” Proceedings of the National Academy of Sciences of the United States of America, vol. 94, no. 16, p. 8539–8544, 1997.

[70] A. Kunwar, S. K. Tripathy, J. Xu, M. K. Mattson, P. Anand, R. Sigua, M. Vershinin, R.J. McKenney, C. C. Yu , A. Mogilner and S. P. Gross , “Mechanical stochastic tug-of-war models cannot explain bidirectional lipid-droplet transport,” Proceedings of the National Academy of Sciences of the United States of America, vol. 108, no. 47, p. 18960–18965, 2011.

[71] M. C. Uçar and R. Lipowsky, “Collective Force Generation by Molecular Motors Is Determined by Strain-Induced Unbinding,” ACS Nano Lett, vol. 20, no. 1, pp. 669-676, 2020.

[72] M. J. Schnitzer, K. Visscher and S. M. Block , “Force production by single kinesin motors,” Nat Cell Biol, no. 2, pp. 718-723, 2000.

[73] K. Svoboda and S. M. Block, “Force and velocity measured for single kinesin molecules,” Cell, vol. 77, no. 5, p. 773–784, 1994.

[74] K. Visscher, M. J. Schnitzer and S. M. Block , “Single kinesin molecules studied with a molecular force clamp,” Nature, vol. 400, pp. 184-189, 1999.

[75] B. H. Blehm, T. A. Schroer, K. M. Trybus, Y. R. Chemla and P. R. Selvin, “In vivo optical trapping indicates kinesin’s stall force is reduced by dynein during intracellular transport,” PNAS, vol. 110, no. 9, pp. 3381-3386, 2013.

[76] A. Marantan, L. Mahadevan, “Mechanics and statistics of the worm-like chain”, Am.J. Phys. 86, 2018.

[77] I. Weber, C. Appert-Rolland, G. Schehr and L. Santen, “Non-equilibrium fluctuations of a semi-flexible filament driven by active cross-linkers”, EPL 120, 38006, 2017.
